# Polymeric Carbon Nitride‐Photocatalyzed Aerobic Epoxidation of Alkenes: Application to a Sustainable Synthesis of Rose Oxide

**DOI:** 10.1002/cssc.202500934

**Published:** 2025-06-16

**Authors:** Felix Lorenz, Thanh Huyen Vuong, Tim Peppel, Jennifer Strunk, Malte Brasholz

**Affiliations:** ^1^ Leibniz‐Institut für Katalyse e.V Albert‐Einstein‐Str. 29a 18059 Rostock Germany; ^2^ Technische Universität München Department Chemie Lichtenbergstr. 4 85748 Garching Germany; ^3^ Universität Rostock Institut für Chemie Albert‐Einstein‐Str. 3A 18059 Rostock Germany

**Keywords:** aerobic oxidation, epoxides, heterogeneous catalysis, photocatalysis, rose oxide

## Abstract

A urea‐based pristine polymeric carbon nitride catalyzes the aerobic epoxidation of citronellol under visible light irradiation with low‐power blue LEDs. Formation of 6,7‐epoxycitronellol in this reaction is remarkably selective yet relatively slow requiring 72 h of continuous irradiation. The aerobic photocatalyzed epoxidation is readily accelerated with isobutyraldehyde as a comediator, and a general photocatalytic procedure for the epoxidation of terpenoid substrates as well as styrene derivatives is thus developed to give the corresponding epoxides in good yields within attractive reaction times. The carbon nitride‐photocatalyzed aerobic epoxidation is demonstrated in 14 examples, and the epoxidation of citronellol enables a short and sustainable three‐step synthesis of the industrially relevant terpenoid fragrance compound rose oxide.

## Graphical abstract

**Heterogenous photocatalyzed alkene epoxidation:** A simple urea‐based polymeric carbon nitride catalyzes the aerobic epoxidation of citronellol and a range of other alkenes under visible light irradiation. Mediation by an aliphatic aldehyde can accelerate the reaction, and the photocatalytic method enables an efficient short synthesis of the terpenoid fragrance compound rose oxide.

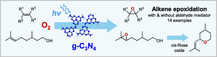

## 1 Introduction

Polymeric carbon nitrides (pCNs) of the formula C_3_N_4_ are a class of photoactive metal‐free semiconductors that are readily accessible from a range of low molecular precursors. The materials possess optical bandgaps of typically around 2.5–2.7 eV, however, their specific surface areas can be considered relatively small in comparison with more common metal chalcogenide and metal pnictogenide semiconductor materials.^[^ ^1^ ^]^ During the last years, many applications of pCNs and of their modified variants in photocatalytic transformations have been introduced. These include CO_2_ photoreduction^[^ ^2^ ^]^ and water splitting,^[^ ^3^ ^]^ the photodegradation of pollutants,^[^ ^4^ ^]^ as well as applications in single electron transfer (SET) induced organic radical reactions.^[^ ^5^ ^]^

Lately, the application of pCNs as photocatalysts in selective aerobic oxidation reactions of organic compounds has gained increasing attention, and a variety of such reactions have been achieved,^[^ ^6^ ^]^ including recently highlighted applications, such as decarboxylative C,H‐oxygenations of carboxylic acids^[^ ^7^, ^8^ ^]^ as well as of *N*‐heterocyclic compounds.^[^ ^9^ ^]^ Further, oxidations of chalcogen‐containing compounds have been described, for instance of organosulfides.^[^ ^6^ ^]^

One important and sustainable type of aerobic oxidation reaction of organic compounds is the epoxidation of alkenes with dioxygen, and over the years, a large variety of homogeneous and heterogeneous metal‐based catalysts have been developed for this purpose.^[^ ^10^ ^]^ In the area of heterogeneous photocatalysis, recent progress in aerobic epoxidation of alkenes has also encompassed the utilization of carbon nitride catalysts, however, so far only epoxidation reactions using either metal‐containing^[^ ^11^ ^]^ or deeply surface‐modified^[^ ^12^ ^]^ variants of pCN materials have been disclosed.

**Scheme 1:**
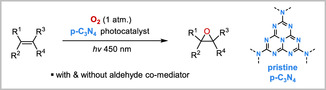
Photocatalytic aerobic oxidation of alkenes with pristine *p*‐C_3_N_4_.

Distinct forms of pristine pCNs are easily made through convenient synthetic protocols, whereby each of these photoactive materials possesses subtle differences in their morphologies and electronic properties.^[^ ^13^ ^]^ In a previous report, we assessed a diverse set of pristine pCNs in several model organic radical transformations, to identify a simple urea‐based pCN as a versatile and reliable catalyst.^[^ ^14^ ^]^ Herein, we wish to disclose that the same urea‐derived pCN catalyst is effective in the aerobic epoxidation of alkenes with dioxygen. While the epoxidation of citronellol as a model substrate occurred selectively despite relatively slow conversion, the same epoxidation was accelerated with co‐mediation by isobutyraldehyde. To our knowledge, this is the first report of an aliphatic aldehyde*‐co*‐mediated pCN‐photocatalyzed epoxidation that occurs with a pristine pCN semiconductor as the catalyst, and the reaction has been demonstrated for terpene, styrene, and stilbene‐type substrates (**Scheme** **1**).

## 2 Results and Discussion

### 2.1 Catalyst Preparation and Reaction Development

Among the pCN semiconductor photocatalysts utilized in this study, a first catalyst material was made by melamine polymerization under N_2_‐atmosphere at 600 °C followed by surface oxidation with HNO_3_/H_2_SO_4_ at ambient temperature for 16 h. This oxidized pCN catalyst, termed **pCN‐Ox** herein, was previously described as an efficient generator of singlet molecular oxygen (^1^O_2_) while irradiated in the presence of ambient oxygen, as a consequence of an improved triplet exciton yield compared to unmodified carbon nitrides.^[^ ^15^, ^16^ ^]^ A second, yet pristine pCN catalyst **pCN‐Ur** was derived from dry urea heated at 550 °C, and this material, which we characterized in depth earlier,^[^ ^14^ ^]^ showed a relatively large specific surface area of ≈54 m^2^ g^−1^ combined with a bandgap of 2.68 eV.

(±)‐Citronellol (**1**) was chosen as a model substrate for an aerobic photocatalytic oxidation with both heterogeneous photocatalysts and the results are summarized in **Table** **1** (for further details, see the Table S1, Supporting Information). To begin with, the homogeneous visible light‐driven photooxygenation of citronellol (**1**) was performed, with methylene blue as a photosensitizer, in MeCN solvent and under 525 nm LED irradiation (18 W, nominal irradiance 13 mW cm^−2^, reactor setup shown in Figure S1, Supporting Information), which cleanly gave the allylic hydroperoxides **2** (*d.r.* 1.8:1) and **3** in near‐quantitative yield within 2 h reaction time (entry 1). Thereafter, the heterogeneously catalyzed version of the reaction was attempted and to our surprise, both pCN catalysts would generate only trace amounts of the hydroperoxides **2** and **3**. Using **pCN‐Ox** as well as the pristine **pCN‐Ur**, each in a quantity of 5 mg per 0.2 mmol of substrate **1**, under 1 atmosphere of O_2_ and 450 nm blue LED irradiation (18 W, 34 mW cm^−2^), produced the unexpected 6,7‐epoxycitronellol **4** (*d.r.* 1:1) as the main product, and almost exclusively in the case of catalyst **pCN‐Ur** (entries 2 and 3). While the formation of epoxide **4** was relatively slow, both catalysts showed comparable conversions of 61%–76% after 72 h, and yields of product **4** of 23% and 30%, respectively. Catalyst loading, excitation wavelength, and reaction duration were subsequently further optimized for the catalyst **pCN‐Ur** (entries 4–6), to result in a procedure that would generate epoxide **4** in 57% yield after 72 h of continuous 450 nm excitation (entry 4). By comparison, use of a 380 nm LED (18 W, 8 mW cm^−2^) resulted in a faster but slightly less selective conversion (entry 6). No reaction occurred in the absence of the photocatalyst or in the dark (see Table S1, Supporting Information). As such, the aerobic photocatalytic epoxidation of citronellol (**1**) with **pCN‐Ur** is a remarkable reaction since **pCN‐Ur** is in fact a pristine, that is, unmodified, carbon nitride material, and the reaction mechanism is intriguing. A number of previous studies utilized only metal‐modified carbon nitride catalysts in aerobic epoxidation reactions.^[^ ^11^ ^]^

**Table 1:** Reaction development of the pCN‐photocatalyzed aerobic epoxidation reaction of (±)‐citronellol (1).

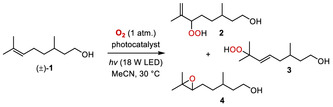						
#	catalyst (mg/0.2 mmol **1**)	additive (eq.)	λ_ex_ [nm]	t [h]	Conva) of **1** [%]	yielda) **2**/**3**/**4** [%]
1	Meth. blue (2)	none	525	2	100	54/45/0
2	pCN‐Ox (5)	none	450	72	61	7/5/25
3	pCN‐Ur (5)	none	450	72	76	0/2/30
**4**	**pCN‐Ur (10)**	**none**	**450**	**72**	**100**	**0/0/57**
5	pCN‐Ur (10)	none	450	48	59	0/1/13
6	pCN‐Ur (10)	none	380	48	87	0/4/34
7	pCN‐Ur (10)	*i*‐PrCHO (1.0)	450	42	98	0/2/75
8	pCN‐Ur (10)	*i*‐PrCHO (2.0)	450	20	98	0/3/77
**9**	**pCN‐Ur (10)**	***i*** **‐PrCHO (3.0)**	**450**	**18**	**100**	**0/2/86**

Conditions: 0.20 mmol alkene, O_2_ (1 atm), photocatalyst, MeCN, LED (18 W), 30 °C.

a) Determined by ^1^H‐NMR against CH_2_Br_2_ standard.

Although with this achievement, a procedure for a selective photocatalytic aerobic epoxidation of citronellol (**1**) with **pCN‐Ur** had been established, the relatively low reaction rate could not be further improved. For this reason, we also studied a Mukaiyama‐type aerobic epoxidation,^[^ ^17^ ^]^ with **pCN‐Ur** as the photocatalyst, and with isobutyraldehyde as a co‐mediator (entries 7–9). These reaction conditions led to a substantially more rapid formation of epoxide **4**, which was obtained in 86% yield, after 18 h of 450 nm irradiation in the presence of 3 molar equivalents of *i*‐PrCHO (entry 9). An earlier report described an example of a heterogenous photocatalyzed aerobic epoxidation of alkenes involving a pCN catalyst in combination with an aliphatic aldehyde mediator. However, therein, again a highly specialized metal‐modified catalyst was employed.^[11c]^ This study demonstrated that simple pristine carbon nitride catalysts, like **pCN‐Ur**, can give in fact excellent results, and control experiments showed that both the semiconductor catalyst and irradiation are essential for product formation in this epoxidation procedure including *i*‐PrCHO (Table S1, Supporting Information).

**Figure 1:**
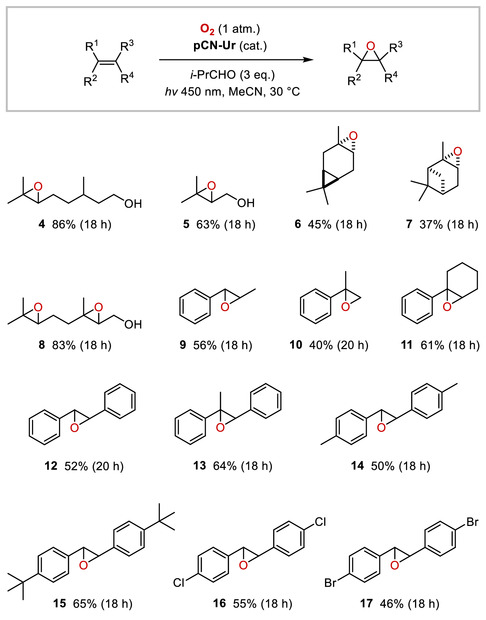
Scope of the photocatalytic aerobic epoxidation reaction. Conditions: 0.20 mmol of alkene, O_2_ (1 atm), **pCN‐Ur** (10 mg per 0.20 mmol of alkene), MeCN, 450 nm LED (18 W, 34 mW·cm^−2^), 30 °C. Yields determined by ^1^ H‐NMR against CH_2_Br_2_ standard, accuracy ±3%.

A scope of the isobutyraldehyde co‐mediated aerobic epoxidation of alkenes photocatalyzed by **pCN‐Ur** is shown in **Figure** **1**. As in the case of citronellol (**1**), several further terpenoids were readily converted into their epoxides. Prenol, (±)‐3‐carene, and (±)‐α‐pinene gave the corresponding epoxides **5–7** in 37%–63% yield after 18 h reaction time. In addition, (±)‐geraniol was converted into diepoxygeraniol **8** in 83% yield, as a mixture of diastereoisomers. Further, styrene derivatives were found to be suitable substrates. *β*‐ And *α*‐methylstyrenes could be converted into their mono‐oxides **9** and **10** in 56% and 40% yield, while 1‐phenylhexene gave rise to epoxide **11** in 61% yield. *trans*‐Stilbene, various di‐*para*‐substituted *trans*‐stilbenes as well as *trans*‐α‐methylstilbene gave the epoxides **12–17** in good yields of 46%–65% after 18‐20 h of irradiation. In the latter examples, typical side products were the aryl aldehydes and benzoic acids resulting from oxidative cleavage of the stilbene C—C double bond, and they were present in around 20%–25% of the total yield. Their formation evidently indicated the participation of superoxide as a reactive oxygen species in the reaction mechanism (vide infra). In all of the examples shown in Figure 1, full conversion of the starting material was observed within the designated reaction time. Pure samples of the epoxide products were obtained by flash chromatography (SI, Supporting Information, file), however, due to the instability of the epoxides on silica gel, the reported yields of the epoxidation reactions were determined prior to purification, by ^1^H nuclear magnetic resonance (NMR) analysis, with ±3% accuracy.

The carbon nitride‐catalyzed aerobic photocatalytic epoxidation enabled a short synthesis of the terpenoid rose oxide. Previous syntheses of this industrially relevant fragrance compound mostly relied on the photo‐ or nonphotochemical formation of hydroperoxides **2** and **3** from citronellol, and due to the continued interest in sustainable resource and energy efficient routes to rose oxide, the different established protocols have been assessed and compared in detail with regard to their economic and environmental profiles.^[^ ^18^ ^]^

**Scheme 2:**
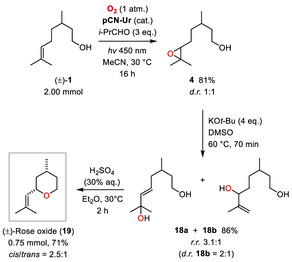
Synthesis of (±)‐rose oxide.

As shown in **Scheme** **2**, (±)‐citronellol (**1**) was successfully converted into epoxide **4** on a 2 mmol scale under co‐mediation by isobutyraldehyde, with 81% yield after 16 h reaction time and chromatographic isolation. Upscaling of the aldehyde‐free photocatalytic epoxidation of citronellol (**1**) with **pCN‐Ur** to 2 mmol was also possible in a larger reaction vial, however, this procedure was complicated by a reduced conversion rate compared to the smaller scale reaction, to result in just 29% of epoxide **4** after 96 h of irradiation. Epoxide **4** (*d.r.* 1:1) was treated with KO*t*‐Bu (4 equiv.) in DMSO at 70 °C,^[^ ^19^ ^]^ to produce the 1,7‐diol **18a** in mixture with 1,6‐diol **18b** (*d.r.* 2:1), in a regioisomer ratio of 3.1:1, and in 86% yield after purification. The inseparable mixture of diols **18a** and **18b** was then subjected to acid‐mediated cyclization with aqueous H_2_SO_4_ in Et_2_O^[^ ^20^ ^]^ at 30 °C, whereupon, the 1,7‐diol **18a** was converted into (±)‐rose oxide (**19**, *cis/trans* 2.5:1) in 71% isolated yield after a final short path distillation. Of note, the three‐step sequence could also be performed without purification of the intermediate products **4** and **18** to deliver (±)‐rose oxide (**19**) in a good 31% of combined yield over three steps, and with a slightly improved diastereoselectivity of 5:1 in favor of the *cis*‐diastereomer (see the SI, Supporting Information).

### 2.2 Discussion of Reaction Mechanisms

**Figure 2:**
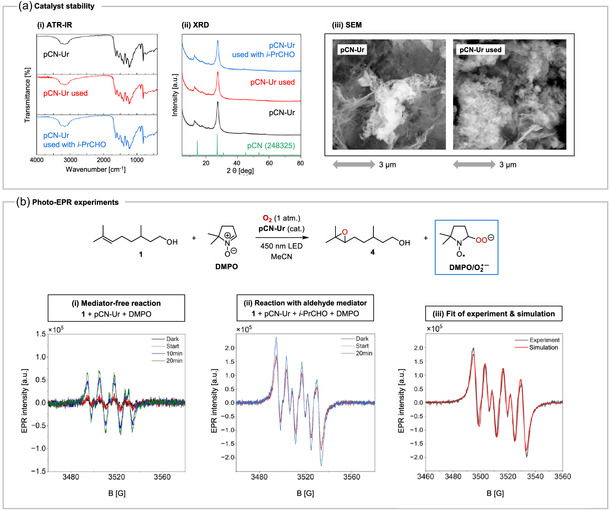
Proof of catalyst stability and photo‐EPR experiments.

First, the catalyst stability under the reaction conditions was assessed and the catalyst **pCN‐Ur** was recollected after the epoxidation of (±)‐citronellol (**1**) with and without added *i*‐PrCHO. **Figure** **2**a (i) shows the attenuated total reflectance‐IR spectra of the catalyst before and after reaction and the material appears to be unchanged after irradiation under the oxidative conditions, showing no appearance or disappearance of bands. The X‐ray diffraction powder diffractograms (ii) also appear to be identical after the reaction, showing no change in crystal structure. Scanning electron microscope (SEM) images (iii) before and after the isobutyraldehyde‐mediated reaction display similar structural features, revealing an essentially unchanged layered morphology (this is also the case after the aldehyde‐free reaction, see Figure S4, Supporting Information). In addition, UV–Vis diffuse reflectance spectra, thermogravimetric analyses, and elemental analyses as well as N_2_ physisorption analyses of the catalyst before and after the reactions were acquired (see Figure S5–S7 and Tables S2 and S3, Supporting Information). These data showed that the oxygen content of **pCN‐Ur** was slightly increased after the reactions as well as its material porosity.

As shown in Figure 2b, electron paramagnetic resonance (EPR) studies were performed to gain insight into the reaction mechanism. A standard reaction mixture with 0.20 mmol of (±)‐citronellol (**1**) and catalytic **pCN‐Ur** was irradiated for 5 h, and then, a sample of the mixture was transferred to an EPR capillary with 5,5‐dimethyl‐1‐pyrroline *N*‐oxide (DMPO) added as a spin trap. For the mediator‐free reaction (i), a characteristic signal for the DMPO/O_2_^•‐^ spin adduct^[^ ^21^ ^]^ was found (A_N_ = 13.77 G, A_H_^β^ = 9.47 G, A_H_^γ^ = 1.24 G), indicating the presence of superoxide (O_2_^•‐^). The intensity of the signal further increased upon additional subsequent in situ irradiation with 456 nm LEDs, substantiating that O_2_^•‐^ was indeed produced photocatalytically. Performing the same experiment with 3 equiv. of *i*‐PrCHO as the aldehyde mediator led to a significant additional increase in signal intensity owing to a further more efficient formation of the DMPO/O_2_^•‐^ adduct. Even exposure to ambient light before the actual sample irradiation was sufficient to yield high signal intensities. Photo‐EPR experiments without catalyst showed only marginal signal intensities, proving **pCN‐Ur** to be the photocatalytic O_2_^•‐^ generator under the reaction conditions (Figure S8, Supporting Information).

**Scheme 3:**
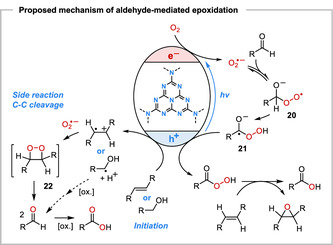
Proposed mechanism of the aliphatic aldehyde‐mediated pCN‐photocatalyzed aerobic epoxidation of alkenes.

A proposed mechanism for the aliphatic aldehyde‐mediated pCN‐photocatalyzed aerobic epoxidation is shown in **Scheme** **3**. Upon electronic excitation of the semiconductor to its charge‐separated [pCN (e^−^/h^+^)]* state, the photogenerated holes (valence band maximum ≈ +1.6 V vs. Standard hydrogen electrode (SHE) are initially quenched by SET from the organic substrate, which based on redox potentials is exergonic for electron‐rich alkenes, like styrenes and stilbenes. Terpenoids, such as citronellol, prenol, and geraniol, with higher oxidation potentials (*E*_ox_ for the C6,C7 double bond of (**1**) ≈ +1.9 V vs. SHE)^[^ ^22^ ^]^ may react via alcohol oxidation to the corresponding aldehydes,^[^ ^23^ ^]^ which are detected in the experiments in small amounts after NMR analysis. The simultaneous SET reduction of molecular oxygen generates superoxide (O_2_^•‐^) as the key reactive oxygen species. Reactions of superoxide radical anion with aliphatic aldehydes in aprotic solvents tend to be unselective,^[^ ^24^ ^]^ however, in the presence of the pCN catalyst, the only detectable byproduct derived from *i*‐PrCHO after the reactions discussed herein is isobutyric acid. O_2_^•‐^ Radical anion generally displays only low reactivity in hydrogen abstraction reactions, and hydrogen atom transfer (HAT) between superoxide and ldehydes to produce an acyl radical is highly endergonic based on bond dissociation energies.^[^ ^25^ ^]^ Consistently, when the model epoxidation reaction was conducted in the presence of TEMPO (1 equiv.), none of the acyl radical trapping product, 2,2,6,6‐tetramethyl‐1‐piperidinyl 2‐methylpropanoate was formed. Therefore, it is proposed that superoxide exerts nucleophilic reactivity and its addition to the aldehyde gives a peroxyl acyl radical anion intermediate **20** in equilibrium.^[^ ^26^ ^]^ A 1,3‐hydrogen shift leads to peroxyl ketyl radical anion **21**, and recent computational results^[^ ^9^ ^]^ suggest that this may be assisted by the semiconductor surface via the formation of intermediate hydrogenated carbon nitride radicals [pCN+H]^•^. Subsequent hole‐mediated SET oxidation of species **21**, which is reminiscent of the known pCN‐catalyzed oxidative esterification of aldehydes with alcohols,^[^ ^27^ ^]^ leads to the percarboxylic acid, to induce the epoxidation of the alkene substrate. For the isobutyraldehyde‐mediated epoxidation of citronellol (**1**), an approximate quantum yield of *Φ* ≈ 2.5 × 10^−4^ was determined, which rules out the contribution of chain propagation reactions.

The epoxidation of *trans*‐stilbene in the presence of *i*‐PrCHO gave *trans*‐stilbene oxide (**12**) accompanied by 10% of benzaldehyde and 18% of benzoic acid. Their formation can be rationalized by the side reaction pathway shown in Scheme 3. SET oxidation of *trans*‐stilbene by photoexcited pCN is well‐possible based on redox potentials (*E*_ox_ of *trans*‐stilbene is +1.39 V vs. SHE).^[^ ^28^ ^]^ The radical cation derived from *trans*‐stilbene then combines with superoxide to give a transient 1,2‐dioxetane intermediate **22**, which collapses to benzaldehyde to be further oxidized to benzoic acid eventually. A third conceivable pathway may be initiated by the autoxidation of the aliphatic aldehyde with molecular oxygen, to generate an acyl peroxyl radical as an active epoxidizing reagent.^[^ ^29^ ^]^ Probing the *i*‐PrCHO‐mediated epoxidation of several substrates shown in Figure 1 in the absence of catalyst **pCN‐Ur** however showed that only trace amounts of the epoxide products were formed within similar reaction times and experimental conditions, hence, the aldehyde autoxidation pathway is not significant when the carbon nitride catalyst is present.

**Scheme 4:**
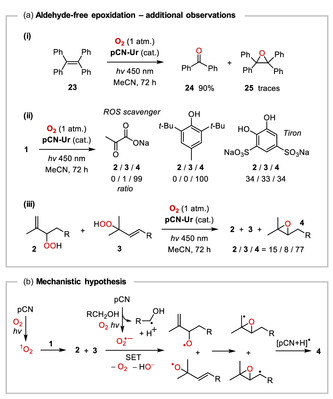
Mechanism‐relevant observations and preliminary mechanistic hypothesis for the aldehyde‐free pCN‐photocatalyzed aerobic epoxidation of citronellol (**1**).

With regard to the isobutyraldehyde‐free photocatalytic aerobic epoxidation of citronellol (**1**) with **pCN‐Ur**, a quantum yield of *Φ* ≈ 4.2 × 10^−5^ was determined, and no byproducts arising from oxidative cleavage of the C6,C7‐double bond were found. Several relevant observations were made in control experiments as shown in **Scheme** **4**. The optimized reaction conditions that gave 6,7‐epoxycitronellol **4** in 57% yield after 72 h (Table 1, entry 4) were much less suitable for other substrates. *trans*‐Stilbene for instance was fully converted after 24 h, however only 14% of epoxide **12** was obtained, along with 31% of benzoic acid, which resulted from oxidative cleavage of the stilbene C—C double bond. Consistently, tetraphenylethene (**23**) was converted to benzophenone (**24**) in 90% yield under the same conditions, while only trace amounts of epoxide **25** could be detected (Scheme 4a, [i]).

The photooxidation of citronellol (**1**) with **pCN‐Ur** was probed in the presence of various reactive oxygen species (ROS) scavenging agents (Scheme 4a, [ii]). With sodium pyruvate (1 equiv.) as an H_2_O_2_ scavenger, epoxide **4** formed cleanly, indicating that H_2_O_2_ is not involved in the conversion of the alkene. This was further corroborated by a reaction of **1** that was performed under Argon atmosphere and with 1 equiv. of H_2_O_2_ instead of oxygen. In this experiment, only 11% conversion of citronellol (**1**) was observed after 72 h, but products **2–4** could not be detected, clearly ruling out H_2_O_2_ as the active oxidant. When the epoxidation of citronellol (**1**) was performed in the presence of butylated hydroxytoluene (BHT) as a hydroxyl radical scavenger, the selectivity toward formation of epoxide **4** similarly remained unaffected. On the contrary, using Tiron (disodium 4,5‐dihydroxybenzene‐1,3‐disulfonate, 1 equiv.), a scavenger of superoxide,^[^ ^30^ ^]^ the formation of epoxide **4** was much less selective, and after 72 h, epoxide **4** was accompanied by equal portions of the allylic hydroperoxides **2** and **3**. This result suggested that when superoxide is quenched, and therefore, present only in a minute concentration, singlet oxygen, which apparently does form by triplet EnT but with very low quantum efficiency, suddenly can compete, to induce a ^1^O_2_ Ene reaction of the alkene **1**. Schenck Ene reactions of alkenes with allylic hydrogens are known to occur with exceptionally high rates,^[^ ^31^ ^]^ and therefore, potentially could outcompete intermolecular reactions between alkenes and superoxide (O_2_^•‐^). In all of the aerobic photooxidations of citronellol (**1**) with catalyst **pCN‐Ox**, the allylic hydroperoxides **2** and **3** were observed in small amounts after 72 h (Table 1, entry 2 and Table S1, Supporting Information), and also using catalyst **pCN‐Ur**, compound **3** could be detected after 72 h (Table 1, entry 3). However, after 96 h reaction time with catalyst **pCN‐Ox**, only epoxide **4** was found in 74% yield (Table S1, Supporting Information). These observations suggest that hydroperoxides **2** and **3** form initially in the reactions of citronellol (**1**) with both catalysts, to be consumed subsequently during the reaction. As shown in Scheme 4a (iii), when the hydroperoxides **2** and **3** in equimolar ratio were subjected to the typical reaction conditions in the presence of **pCN‐Ur**, they were largely converted into epoxide **4** (the ratio **2**/**3/4** after 72 h was 15/8/77 by ^1^H‐NMR analysis). By comparison, irradiation of compounds **2** and **3** without **pCN‐Ur** for the same duration only showed around 20% conversion to epoxide **4**.

The collective observations currently do not provide a conclusive mechanistic picture for the pCN‐catalyzed aldehyde‐free aerobic epoxidation of citronellol (**1**). As a preliminary hypothesis, it is suggested that initially a singlet oxygen Ene reaction of **1** occurs, and that the O—O bond of hydroperoxides **2** and **3** is then cleaved with the assistance of superoxide radical anion, the generation of which is coupled to the sacrificial oxidative reaction of alcohol **1** (Scheme 4b). HO^‐^ formed during the reaction can serve as additional electron donor. The SET reduction of hydroperoxides **2** and **3** by _2_^•^O^‐^, which can be regarded as a Haber–Weiss type reaction^[25a]^ would produce the corresponding alkoxyradicals. Their 3‐exo radical cyclizations followed by HAT, likely from hydrogenated pCN surface sites [pCN + H]^•^, convergently produce epoxide **4**.

## 3 Conclusion

In this study, pristine urea‐based pCN was utilized as a selective photocatalyst for the aerobic epoxidation of various terpenoids, styrenes, and stilbenes, using *i*‐PrCHO as a co‐mediator for improved yields and shorter reaction times. Upscaled epoxidation of (±)‐citronellol (**1**) enabled the three‐step synthesis of (±)‐rose oxide (**19**) with 31% overall yield, following an unconventional, sustainable synthetic route. High stability of pCN under the reaction conditions was proven through multiple characterization methods. In situ photo‐EPR measurements confirmed the involvement of superoxide in the *i*‐PrCHO‐mediated reaction, substantiating a Mukaiyama‐type epoxidation mechanism. The aldehyde‐free epoxidation was investigated by means of in situ photo‐EPR, ROS scavenging, and additional control experiments, leading to a preliminary mechanistic rationale.

## Conflict of Interest

The authors declare no conflict of interest.

## Supporting information

Supplementary Material

## Data Availability

The data that support the findings of this study are available in the supplementary material of this article.
