# Supplementary material for: Polymeric Carbon Nitride‐Photocatalyzed Aerobic Epoxidation of Alkenes: Application to a Sustainable Synthesis of Rose Oxide
Source: ChemSusChem. 2025 Jun 16;18(15):e202500934. doi: 10.1002/cssc.202500934 (PMC12302317; doi:10.1002/cssc.202500934)
Supplement: Supplementary file 1 — Supplementary Material [file CSSC-18-e202500934-s001.pdf]

Supporting Information

Polymeric carbon nitride-photocatalyzed aerobic epoxidation of alkenes: Application to a sustainable synthesis of Rose oxide

Felix Lorenz,[a] Thanh Huyen Vuong,[a] Tim Peppel,[a]
Jennifer Strunk,*[a,b] Malte Brasholz*[a,c]

[a] Leibniz-Institut für Katalyse e.V., Albert-Einstein-Str. 29a, 18059 Rostock, Germany

[b] Technische Universität München, Department Chemie, Lichtenbergstr. 4, 85748 Garching, Germany

[c] University of Rostock, Institute of Chemistry, Albert-Einstein-Str. 3a, 18059 Rostock, Germany.

Email: malte.brasholz@uni-rostock.de; jennifer.strunk@tum.de

Contents:

1 General information ____ SI 2 – SI 3

2 Catalyst syntheses ____ SI 3

3 Reaction development & quantum yields ____ SI 4 – SI 5

4 Catalyst characterization before and after reactions ____ SI 6 – SI 9

5 EPR Experiments ____ SI 10

6 Experimental procedures: epoxidation reactions & synthesis of Rose oxide SI 11 – SI 20

7 NMR Spectra ____ SI 21 – SI 36

8 References ____ SI 37

1 General information

1.1 Synthetic works

Commercially available chemicals were used as received from suppliers unless otherwise noted. Dry solvents used were obtained from suppliers in serum-cap quality. Solvents for chromatographic separation were distilled twice prior to use. Thin-layer chromatography was carried out using silica-coated aluminium plates, silica 60 F254, Merck. Column chromatography was performed with silica 60 (230-400 mesh, Macherey-Nagel). NMR spectra were recorded on Bruker AVANCE 500 NEO and JEOL JNM-ECZ400 instruments, and spectra were calibrated against the solvent resonances of $CHCl_3$ (δ^H = 7.26 ppm) and $CDCl_3$ (δ^C = 77.2 ppm).

Photocatalytic reactions were performed in an EvoluChem™ PhotoRedOx Box with in-built mirror reflectors, using 18 W LEDs as light sources with three different wavelengths: 380 nm (8 mW·cm^{-2} nominal irradiance), 450 nm (34 mW·cm^{-2}) and 525 nm (13 mW·cm^{-2}).

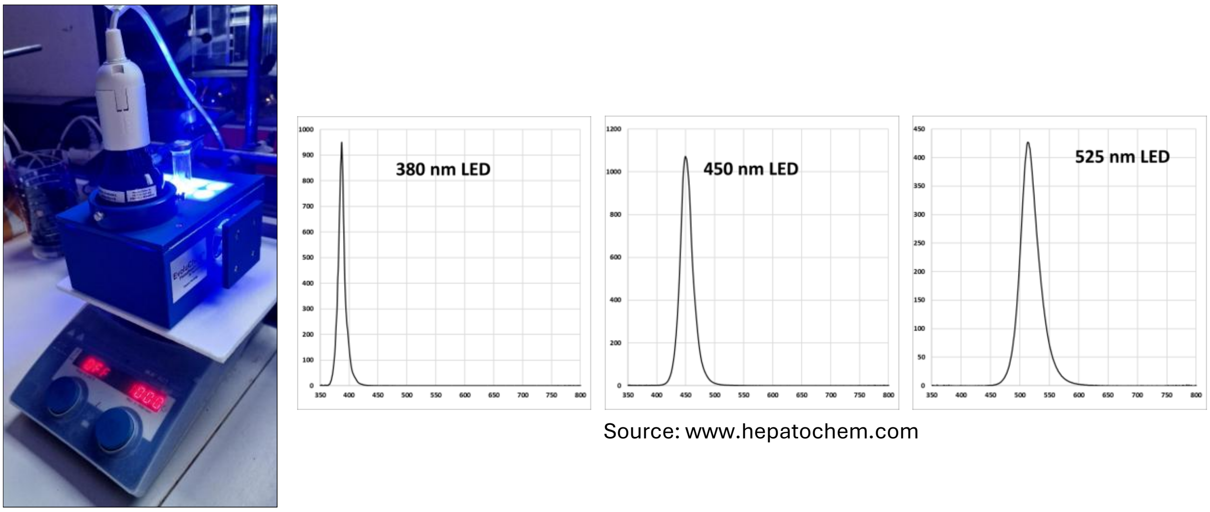

Figure S1. Reaction setup. EvoluChem™ PhotoRedOx Box and LED emission spectra.

1.2 Physical characterization methods

Catalyst morphology was investigated with a Field emission scanning electron microscope (FE-SEM, MERLIN® VP Compact, Co. Zeiss, Oberkochen), equipped with a secondary electron detector (HE-SE2). A Leco TruSpec Micro CHNS device was used to perform elemental analysis (EA). X-ray powder diffraction (XRD) patterns were measured with a Panalytical X'pert Pro powder diffractometer utilizing an X'celerator semiconductor detector, employing Cu K$\alpha_{1,2}$ radiation (40 kV, 40 mA; λ = 0.15406 nm, 0.154443 nm). For the recording of attenuated total reflection infrared (ATR-IR) spectra, a Bruker Alpha FT-IR spectrometer in a spectral range from 400 to 4000 cm^{-1} was employed. Thermogravimetric analysis (TGA) was performed on an STA 449 F5 Jupiter (Netzsch). The sample was heated in synthetic air from room temperature to 600 °C at a heating rate of 10 K/min. UV-Vis diffuse reflectance spectroscopy (UV-Vis DRS) was measured using an integrating sphere installed in a Lambda 365 UV/Vis spectrophotometer (PerkinElmer) in a photometric range of 200-1100 nm at room

temperature. After degassing in vacuum at 200 °C, N_2 physisorption was measured using a NOVAtouch (Anton Paar). Obtained Isotherms were used to calculate the specific surface area of catalysts based on the BET method and the pore size distribution based on the BJH method, as well as the total pore volume and the average pore size.

2 Catalyst syntheses

2.1 Synthesis of pCN-Ox

Following the literature procedure,[1] a 30 mL ceramic crucible was filled with 10 mg of melamine and covered with a lid. In a tube furnace the crucible was heated to 600 °C for 10 h with a heating rate of 3 K/min. The final product was ground in a mortar, resulting in 4,44 g of pristine pCN as a yellow powder. 1 g of pristine pCN was added to a nitrating mixture, containing concentrated H_2SO_4 and HNO_3 in a volumetric ratio of 1:2 and stirred overnight. The mixture was then poured into 300 mL of H_2O and the catalyst was collected by means of filtration. After washing with H_2O for several times and drying in air at room temperature, 803 mg of pCN-Ox were obtained as a yellow powder.

2.2 Synthesis of pCN-Ur

Following the literature procedure,[2] a 60 mL ceramic crucible was filled with 15 g of urea and covered with a lid. In a muffle furnace the crucible was heated to 550 °C for 4 h in air with a heating rate of 2 K/min. The final product was ground in a mortar, resulting in 643 mg of pCN-Ur as a light-yellow, voluminous powder.

3 Reaction development & quantum yields

3.1 Table S1. Reaction development and blind experiments for the photocatalyzed aerobic epoxidaton of (±)-citronellol (**1**).

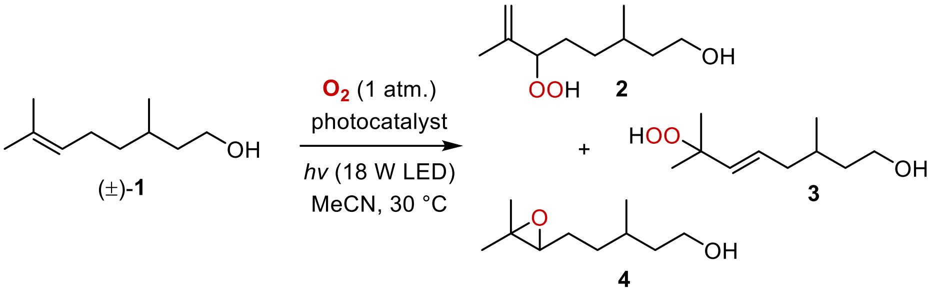

#	catalyst (mg/0.2 mmol **1**)	solvent	additive (eq.)	$λ_{ex}$ [nm]	t [h]	conv. of **1** [%][a]	yield **2** / **3** / **4** [%][a]
1	-	MeCN	none	450	72	1	0 / 0 / 0
2	Methylene blue (2)	MeCN	none	525	2	100	54 / 45 / 0
3	pCN-Ox (5)	MeCN	none	-	72	5	0 / 0 / 0
4	pCN-Ox (25)	MeCN	Ar-atmosphere	450	72	11	0 / 0 / 0
5	pCN-Ox (5)	MeCN	none	450	72	61	7 / 5 / 25
6	pCN-Ox (25)	MeCN	none	450	96	100	0 / 0 / 74
7	pCN-Ur (5)	MeCN	none	450	72	76	0 / 2 / 30
**8**	**pCN-Ur (10)**	MeCN	**none**	**450**	**72**	**100**	**0 / 0 / 57**
9	pCN-Ur (15)	MeCN	none	450	72	100	0 / 0 / 58
10	pCN-Ur (20)	MeCN	none	450	72	100	0 / 0 / 50
11	pCN-Ur (10)	MeCN	none	450	48	59	0 / 1 / 13
12	pCN-Ur (10)	MeCN	none	380	48	87	0 / 4 / 34
13	pCN-Ur (10)	ethyl acetate	none	450	72	54	0 / 0 / 26
14	pCN-Ur (10)	ethanol	none	450	72	56	0 / 2 / 13
15	pCN-Ur (10)	toluene	none	450	72	55	0 / 0 / 22
16	pCN-Ur (10)	MeCN	*i*-PrCHO (0.5)	450	72	94	0 / 0 / 73
17	pCN-Ur (10)	MeCN	*i*-PrCHO (1.0)	450	42	98	0 / 2 / 75
18	pCN-Ur (10)	MeCN	*i*-PrCHO (2.0)	450	20	98	0 / 3 / 77
**19**	**pCN-Ur (10)**	MeCN	***i*-PrCHO (3.0)**	**450**	**18**	**100**	**0 / 2 / 86**
20	pCN-Ur (10)	MeCN	*i*-PrCHO (5.0)	450	18	100	0 / 0 / 93
21	-	MeCN	*i*-PrCHO (3.0)	450	18	12	0 / 1 / 5
22	-	MeCN	*i*-PrCHO (3.0)	-	18	12	0 / 0 / 2

Conditions: 0.20 mmol alkene, O_2 (1 atm), photocatalyst, MeCN, LED (18 W), 30 °C. a) Determined by ^{1}H-NMR against CH_2Br_2 standard.

3.2 Calculation of estimated quantum yields

The actual irradiance, inside the cavity of the reactor setup in Figure S1, equipped with the 450 nm LED lamp, was measured using an electronic light meter, to be

$E_e = 200\ \mathrm{mW} / \mathrm{cm}^2 = 2000\ \mathrm{J} / \mathrm{s} \times \mathrm{m}^2$

Using the 10 mL crimp cap vial as standard reaction vessel ($\mathrm{r} = 0.80\ \mathrm{cm}$), with a $2.00\ \mathrm{mL}$ volume of the reaction mixture, the irradiated surface area is

$(5\ \mathrm{cm} \times 2\ \mathrm{cm}) + (4\pi \times \mathrm{r}^2) / 2 = 14.00\ \mathrm{cm}^2 = 0.0014\ \mathrm{m}^2$

According to $E = \mathrm{h} \times \mathrm{c} / \lambda$, the 450 nm photon energy is $E = 4.414 \times 10^{-19}\ \mathrm{J}$

The radiant flux is $\Phi_e = (2000\ \mathrm{J} / \mathrm{s} \times \mathrm{m}^2) \times 0.0014\ \mathrm{m}^2 = 2.8\ \mathrm{J} / s$

The number of moles of photons per second is

$$N\ (\mathrm{mol} / \mathrm{s}) = \frac{2.8\ \mathrm{J}}{4.414 \times 10^{-19} \times 6.022 \times 10^{23}\ \mathrm{J} \times s} = 1.053\ \times\ 10^{-5}$$

a) Quantum yield for Table S1, entry 8: $$\Phi = \frac{2 \times 10^{-4}\ \mathrm{mol} \times 0.57 \times \mathrm{s}}{259200\ \mathrm{s} \times 1.053 \times 10^{-5}\ \mathrm{mol}} = 4.18\ \times\ 10^{-5}$$

b) Quantum yield for Table S1, entry 19: $$\Phi = \frac{2 \times 10^{-4}\ \mathrm{mol} \times 0.86 \times \mathrm{s}}{64800\ \mathrm{s} \times 1.053 \times 10^{-5}\ \mathrm{mol}} = 2.52\ \times\ 10^{-4}$$

4 Catalyst characterization before and after reactions

Analyses of the catalyst **pCN-Ur** were performed before and after the aerobic photoepoxidation of citronellol (**1**), for each epoxidation procedure, without and with *i*-PrCHO as aldehyde mediator.

4.1 ATR-IR spectra

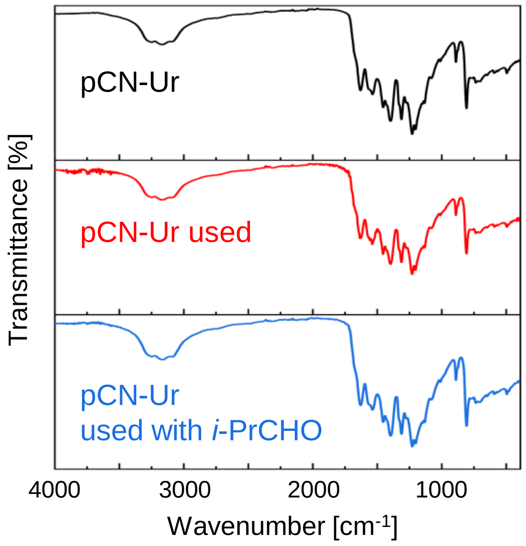

Figure S2. ATR-IR spectra of the catalyst **pCN-Ur** before and after reactions. The data for the unused **pCN-Ur** alone appeared previously. Black graph in image taken from reference [3] under the Creative Commons CC BY-SA 4.0 license.

4.2 XRD powder diffractograms

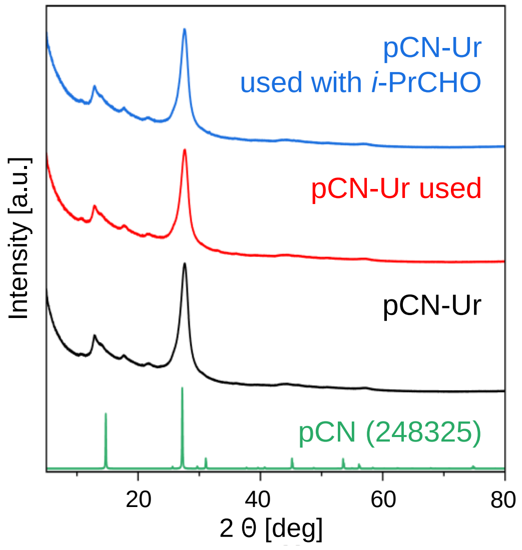

Figure S3. XRD powder diffractograms of the catalyst **pCN-Ur** before and after reactions. The data for the unused **pCN-Ur** alone appeared previously. Black graph in image taken from reference [3] under the Creative Commons CC BY-SA 4.0 license.

4.3 SEM Images

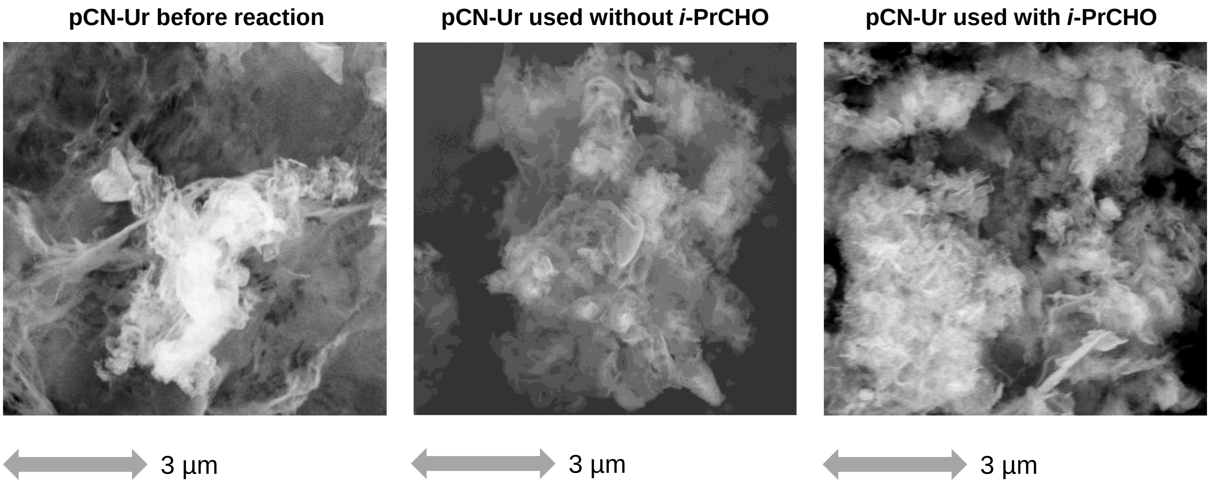

Figure S4. SEM Images of the catalyst **pCN-Ur** before and after reactions. Comparable image data for the unused **pCN-Ur** alone appeared previously.[3]

4.4 Ultraviolet-Visible Diffuse Reflectance Spectroscopy (UV-Vis DRS)

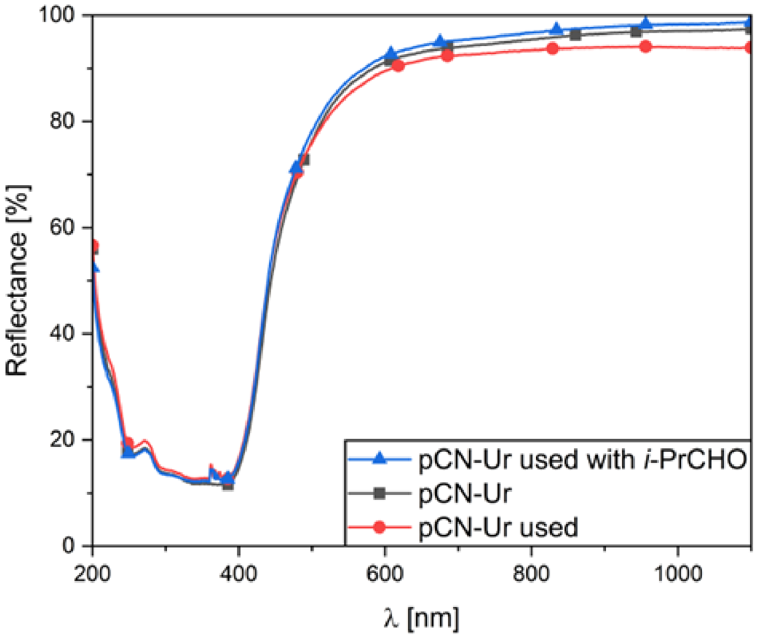

Figure S5. UV-Vis DRS spectra of **pCN-Ur** before and after reactions. The optical band gap determined by Tauc-plot method remains unchanged at 2.7 eV. The data for the unused **pCN-Ur** alone appeared previously. Black graph in image taken from reference [3] under the Creative Commons CC BY-SA 4.0 license.

4.5 Thermogravimetric Analysis (TGA)

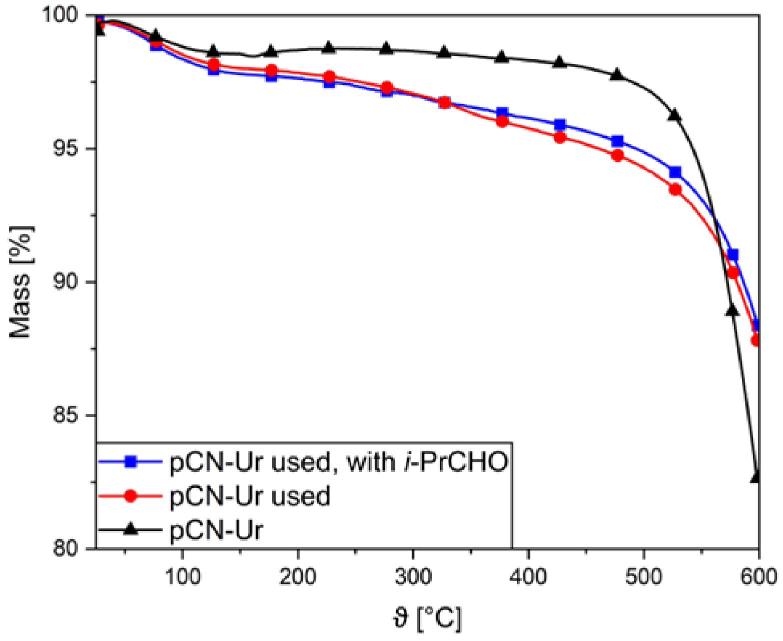

Figure S6. TGA of **pCN-Ur** before and after reactions. The data for the unused **pCN-Ur** alone appeared previously. Black graph in image taken from reference [3] under the Creative Commons CC BY-SA 4.0 license.

4.6 Elemental analyses

Table S2. Elemental analysis of **pCN-Ur** before and after reactions.

element	content in **pCN-Ur** [wt-%]	content in **pCN-Ur** used [wt-%]	content in **pCN-Ur** used, with *i*-PrCHO [wt-%]
C	34.1	33.2	33.0
H	1.1	1.0	0.6
N	58.9	55.9	55.6
O	5.9	9.9	10.8

The data for the unused **pCN-Ur** alone appeared previously. Data taken from reference [3] under the Creative Commons CC BY-SA 4.0 license.

4.7 N_2 Physisorption and Pore Size Distribution

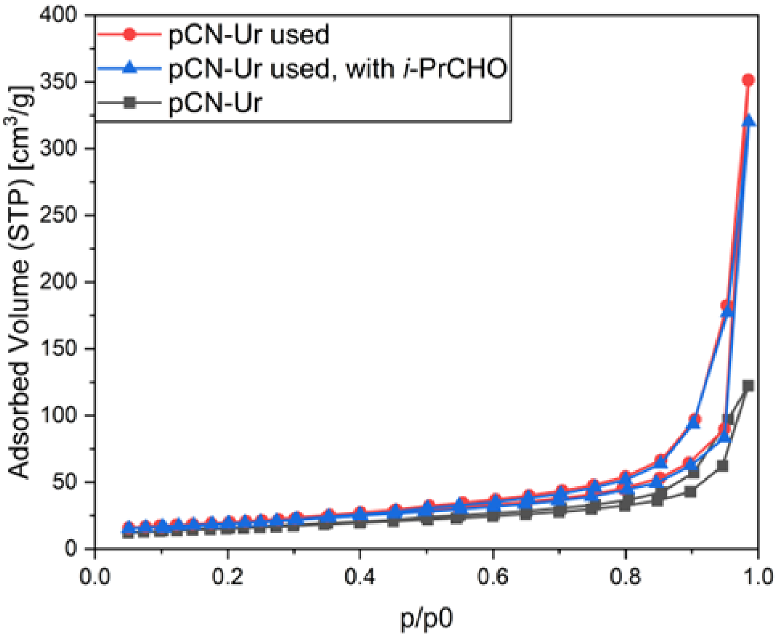

Figure S7. N_2 physisorption isotherms of **pCN-Ur** before and after reactions. The data for the unused **pCN-Ur** alone appeared previously. Black graph in image taken from reference [3] under the Creative Commons CC BY-SA 4.0 license

Table S3. Calculated properties of **pCN-Ur** before and after reactions based on N_2 physisorption isotherms.

material	BET specific surface area [m^2/g]	total pore volume [cm^3/g]	average pore radius [nm]
pCN-Ur	54	0.19	7.1
pCN-Ur used	70	0.54	15.6
pCN-Ur used, with *i*-PrCHO	67	0.50	14.9

The data for the unused **pCN-Ur** alone appeared previously. Data taken from reference [3] under the Creative Commons CC BY-SA 4.0 license.

5 EPR Experiments

EPR spectra were recorded on an X-band Bruker EMX CW-micro EPR spectrometer equipped with an ER4119HS high-sensitivity resonator using a microwave frequency of $\nu \approx 9.7$ GHz, a microwave power of 6.3 mW, a modulation frequency of 100 kHz, a modulation amplitude of 1 G, a scanning number of 1 and sweeping time of 60 s. The $h\nu = g\beta B_0$ equation was used to calculate g values with ν and B_0 being the microwave frequency and resonance field, respectively. 2,2-Diphenyl-1-picrylhydrazyl (DPPH) was used as a standard (g = 2.0036 ± 0.0004) for calibration of the g value. The EPR spectra were simulated with MatlabR2023a using the EasySpin-5.2.36 module.

EPR spin trapping experiments with 5,5-dimethyl-1-pyrroline *N*-oxide (DMPO) were performed as the following: A reaction mixture with 0.20 mmol of (±)-citronellol (**1**) without and with 3 equiv. of *i*-PrCHO as the aldehyde mediator was irradiated for 5 h. About 100 μL of this reaction mixture was taken out and mixed with 10 μL DMPO. Then about 50 μL of this mixture was transferred into a glass microcapillary tube (Hirschmann) and EPR spectra were recorded in dark and under irradiation (40 W Kessil lamp 456nm) at room temperature. EPR experiments were carried out in similar way with a mixture without catalyst.

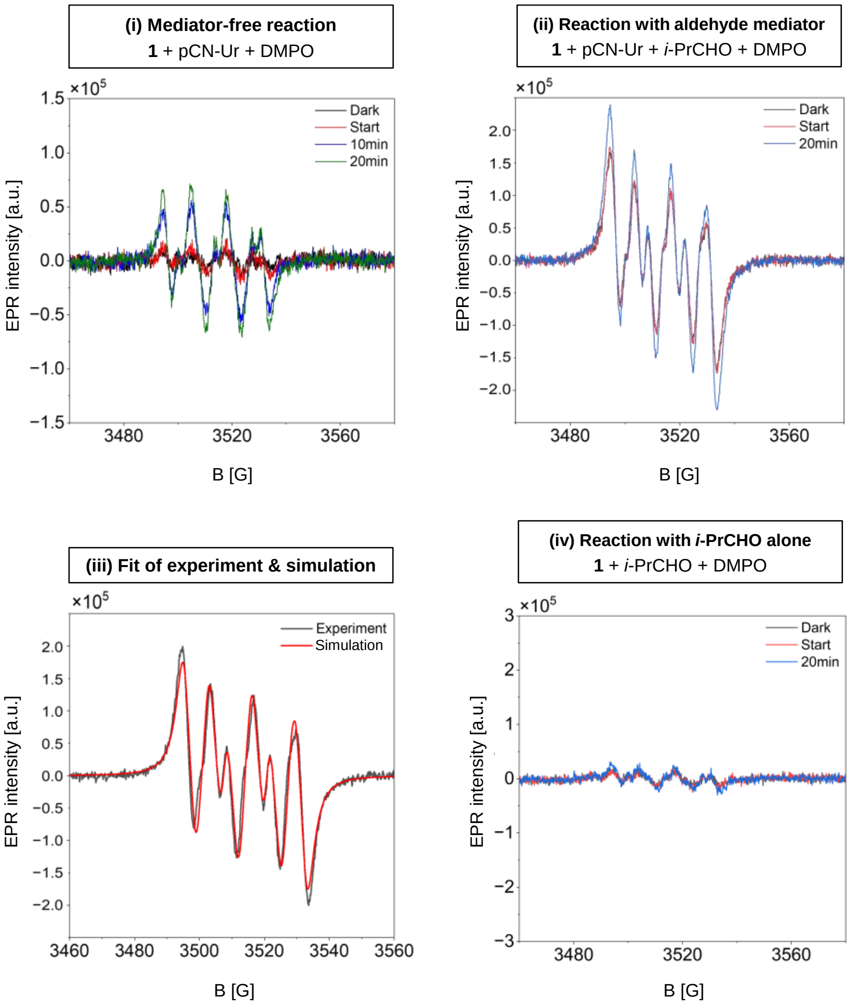

Figure S8. Collected EPR spectra.

6 Experimental procedures: epoxidation reactions & synthesis of Rose oxide

6.1 General Procedure 1: Photocatalytic epoxidation reactions

An oven-dried 10 mL crimp cap vial was charged with photocatalyst **pCN-Ur** (10.0 mg per 0.20 mmol of alkene substrate), dry MeCN (2.00 mL), isobutyraldehyde (0.60 mmol, 54.8 µL, 3 equiv.) and the alkene (0.20 mmol, 1 equiv., resulting concentration of 0.10 M). The vial was sealed and O_2 was bubbled through the mixture via cannula for 10 min. An O_2-filled balloon was fitted via cannula to provide an O_2 atmosphere of 1 atm during the reaction. The reaction mixture was stirred at 30 °C and irradiated for the indicated time (blue LED, *hv* = 450 ± 25 nm, 18 W, 34 mW·cm^{-2}, EvoluChem™ PhotoRedOx Box). After filtration through a PTFE syringe filter, the reaction mixture was washed with a 1 M $Na_2S_2O_3$ solution and extracted with Et_2O (3×). The combined organic layers were dried over Na_2SO_4, filtered, and the solvent was removed under reduced pressure. The crude mixture was analyzed by ^{1}H-NMR spectroscopy using $CDCl_3$ as solvent and CH_2Br_2 as internal standard. Pure samples of the products were obtained by flash column chromatography over silica gel. *Note: Due to the instability of the epoxide products on silica gel, chromatographic isolation was accompanied by some material losses, and thus NMR yields with an accuracy of ±3% are reported.*

(±)-6,7-Epoxycitronellol (4)

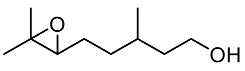

4

Following the general procedure, **pCN-Ur** (10.0 mg), dry MeCN (2.00 mL), isobutyraldehyde (54.8 µL, 0.60 mmol) and (±)-citronellol (**1**, 36.3 µL, 0.20 mmol) after 18 h gave a crude mixture containing compound **4** with a yield of 86% (*d.r.* 1:1), as determined by internal NMR standard. Column chromatography (silica, heptane/EtOAc 2:1 → 1:1) provided the pure product.

^{1}H-NMR (400 MHz, $CDCl_3$): δ = 3.73-3.61 (m, 2 H), 2.71-2.68 (m, 1 H), 1.69-1.46 (m, 5 H), 1.46-1.34 (m, 2 H), 1.29 (s, 3 H), 1.25 (s, 3 H), 0.93-0.90 (m, 3 H) ppm.

^{13}C-NMR (100 MHz, $CDCl_3$): δ = 64.63, 64.61, 60.9, 58.4, 58.3, 39.8, 39.6, 33.71, 33.67, 29.4, 29.2, 26.4, 26.2, 24.9, 19.6, 19.4, 18.7, 18.6 ppm.

Spectroscopic data are in agreement with the literature.[4]

2,3-Epoxy-3-methyl-1-butanol (5)

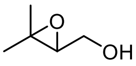

5

Following the general procedure, **pCN-Ur** (10.0 mg), dry MeCN (2.00 mL), isobutyraldehyde (54.8 µL, 0.60 mmol) and prenol (20.3 µL, 0.20 mmol) after 18 h gave a crude mixture

containing compound **5** with a yield of 63%, as determined by internal NMR standard. Column chromatography (silica, pentane/Et_2O 2:1) provided the pure product.

^{1}H-NMR (500 MHz, $CDCl_3$): δ = 3.84 (dd, *J* = 12.1, 4.2 Hz, 1 H), 3.68 (dd, *J* = 12.1, 6.8 Hz, 1 H), 2.98 (dd, *J* = 6.8, 4.3 Hz, 1 H), 1.35 (s, 3 H), 1.31 (s, 3 H) ppm.

^{13}C-NMR (125 MHz, $CDCl_3$): δ = 63.7, 61.5, 58.9, 24.8, 18.8 ppm.

Spectroscopic data are in agreement with the literature.[5]

(±)-3,4-Epoxycarane (6)

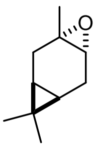

6

Following the general procedure, **pCN-Ur** (10.0 mg), dry MeCN (2.00 mL), isobutyraldehyde (54.8 µL, 0.60 mmol) and (±)-3-carene (31.7 µL, 0.20 mmol) after 18 h gave a crude mixture containing compound **6** with a yield of 45%, as determined by internal NMR standard. Column chromatography (silica, pentane/Et_2O 99:1 → 20:1) provided the pure product.

^{1}H-NMR (500 MHz, $CDCl_3$): δ = 2.82 (t, *J* = 2.0 Hz, 1 H), 2.29 (ddd, *J* = 16.5, 9.2, 2.0 Hz, 1 H), 2.14 (ddd, *J* = 16.1, 9.1, 0.5 Hz, 1 H), 1.63 (dt, *J* = 16.6, 2.3 Hz, 1 H), 1.49 (dd, *J* = 16.1, 2.3 Hz, 1 H), 1.25 (s, 3 H), 1.00 (s, 3 H), 0.72 (s, 3 H), 0.52 (td, *J* = 9.1, 2.3 Hz, 1 H), 0.44 (td, *J* = 9.2, 2.2 Hz, 1 H) ppm.

^{13}C-NMR (126 MHz, $CDCl_3$): δ = 58.2, 55.9, 27.7, 23.3, 23.1, 19.2, 15.99, 15.97, 14.6, 13.8 ppm.

Spectroscopic data are in agreement with the literature.[6]

(±)-2,3-Epoxypinane (7)

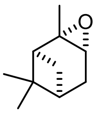

7

Following the general procedure, **pCN-Ur** (10.0 mg), dry MeCN (2.00 mL), isobutyraldehyde (54.8 µL, 0.60 mmol) and (±)-α-pinene (31.7 µL, 0.20 mmol) after 18 h gave a crude mixture containing compound **7** with a yield of 37%, as determined by internal NMR standard. The compound was unstable towards column chromatography.

^{1}H-NMR (500 MHz, $CDCl_3$): δ = 3.06 (dd, *J* = 4.1, 1.1 Hz, 1 H), 2.02-1.86 (m, 4 H), 1.75-1.68 (m, 1 H), 1.59 (d, *J* = 9.5 Hz, 1 H), 1.33 (s, 3 H), 1.27 (s, 3 H), 0.92 (s, 3 H) ppm.

^{13}C-NMR (126 MHz, $CDCl_3$): δ = 60.4, 56.9, 45.0, 40.5, 39.7, 27.6, 26.7, 25.8, 22.4, 20.1 ppm.

Spectroscopic data are in agreement with the literature.[7]

2,3,6,7-Diepoxygeraniol (8)

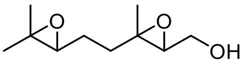

8

Following the general procedure, **pCN-Ur** (10.0 mg), dry MeCN (2.00 mL), isobutyraldehyde (54.8 µL, 0.60 mmol) and geraniol (35.1 µL, 0.20 mmol) after 18 h gave a crude mixture containing compound **8** with a yield of 83% (mixture of diastereomers), as determined by internal NMR standard. Column chromatography (silica, pentane/Et_2O 1:1 → 1:3) provided the pure product.

^{1}H-NMR (400 MHz, $CDCl_3$): δ = 3.85-3.65 (m, 2 H), 3.01-2.97 (m, 1 H), 2.80-2.72 (m, 1 H), 2.46-2.37 (m, 0.6 H), 1.96-1.77 (m, 2 H), 1.59-1.49 (m, 2 H), 1.35-1.26 (m, 9 H) ppm.

^{13}C-NMR (100 MHz, $CDCl_3$): δ = 64.3, 63.8, 62.7, 62.3, 61.3, 60.9, 60.7, 58.8, 58.5, 36.2, 35.1, 24.82, 24.75, 24.5, 18.73, 18.66, 16.9, 16.4 ppm.

Spectroscopic data are in agreement with the literature.[7]

β-Methylstyrene oxide (9)

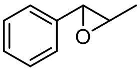

9

Following the general procedure, **pCN-Ur** (10.0 mg), dry MeCN (2.00 mL), isobutyraldehyde (54.8 µL, 0.60 mmol) and β-methylstyrene (25.9 µL, 0.20 mmol) after 18 h gave a crude mixture containing compound **9** with a yield of 56%, as determined by internal NMR standard. Column chromatography (silica, pentane/Et_2O 99:1) provided the pure product.

^{1}H-NMR (400 MHz, $CDCl_3$): δ = 7.37-7.24 (m, 5 H), 3.58 (d, *J* = 2.1 Hz, 1 H), 3.04 (qd, *J* = 5.2, 2.1 Hz, 1 H), 1.46 (d, *J* = 5.2 Hz, 3 H) ppm.

^{13}C-NMR (100 MHz, $CDCl_3$): δ = 137.7, 128.4, 128.0, 125.5, 59.5, 59.0, 17.9 ppm.

Spectroscopic data are in agreement with the literature.[7]

α-Methylstyrene oxide (10)

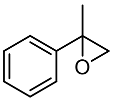

10

Following the general procedure, **pCN-Ur** (10.0 mg), dry MeCN (2.00 mL), isobutyraldehyde (54.8 µL, 0.60 mmol) and α-methylstyrene (25.9 µL, 0.20 mmol) after 20 h gave a crude mixture containing compound **10** with a yield of 40%, as determined by internal NMR standard. Column chromatography (silica, pentane/Et_2O 99:1) provided the pure product.

^{1}H-NMR (400 MHz, $CDCl_3$): 7.39-7.27 (m, 5 H), 2.98 (d, *J* = 5.4 Hz, 1 H), 2.81 (br. d, *J* = 5.4 Hz, 1 H), 1.72 (s, 3 H) ppm.

^{13}C-NMR (100 MHz, $CDCl_3$): δ = 141.2, 128.3, 127.4, 125.3, 57.0, 56.8, 21.8 ppm.

Spectroscopic data are in agreement with the literature.[8]

1,2-Epoxy-1-phenylcyclohexane (11)

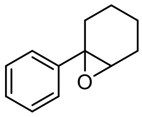

11

Following the general procedure, **pCN-Ur** (10.0 mg), dry MeCN (2.00 mL), isobutyraldehyde (54.8 µL, 0.60 mmol) and 1-phenylcyclohexene (31.8 µL, 0.20 mmol) after 18 h gave a crude mixture containing compound **11** with a yield of 61%, as determined by internal NMR standard. Column chromatography (silica, pentane/Et_2O 99:1) provided the pure product.

^{1}H-NMR (400 MHz, $CDCl_3$): 7.40-7.31 (m, 4 H), 7.29-7.23 (m, 1 H), 3.09-3.07 (m, 1 H), 2.29 (ddd. *J* = 9.1, 8.5, 5.4 Hz, 1 H), 2.16-2.09 (m, 1 H), 2.04-1.97 (m, 2 H), 1.66-1.53 (m, 2 H), 1.52-1.43 (m, 1 H), 1.38-1.28 (m, 1 H) ppm.

^{13}C-NMR (100 MHz, $CDCl_3$): δ = 142.5, 128.2, 127.2, 125.3, 61.9, 60.2, 28.8, 24.7, 20.1, 19.8 ppm.

Spectroscopic data are in agreement with the literature.[7]

trans-Stilbene oxide (12)

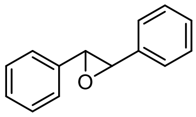

12

Following the general procedure, **pCN-Ur** (10.0 mg), dry MeCN (2.00 mL), isobutyraldehyde (54.8 µL, 0.60 mmol) and *trans*-stilbene (36.0 mg, 0.20 mmol) after 20 h gave a crude mixture containing compound **12** with a yield of 52%, as determined by internal NMR standard. Column chromatography (silica, heptane/EtOAc 98:2) provided the pure product.

^{1}H-NMR (500 MHz, $CDCl_3$): δ = 7.42-7.32 (m, 10 H), 3.88 (s, 2 H) ppm.

^{13}C-NMR (126 MHz, $CDCl_3$): δ = 137.1, 128.6, 128.3, 125.5, 62.8 ppm.

Spectroscopic data are in agreement with the literature.[9]

***trans*-α-Methylstilbene oxide (13)**

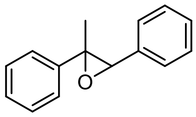

13

Following the general procedure, **pCN-Ur** (10.0 mg), dry MeCN (2.00 mL), isobutyraldehyde (54.8 µL, 0.60 mmol) and α-methylstilbene (38.9 mg, 0.20 mmol) after 18 h gave a crude mixture containing compound **13** with a yield of 64%, as determined by internal NMR standard. Column chromatography (silica, heptane/EtOAc 200:1) provided the pure product.

^{1}H-NMR (500 MHz, $CDCl_3$): δ = 7.47-7.45 (m, 2 H), 7.41-7.36 (m, 6 H), 7.34-7.30 (m, 2 H), 3.98 (s, 1 H), 1.47 (s, 3 H) ppm.

^{13}C-NMR (126 MHz, $CDCl_3$): δ = 142.3, 135.9, 128.4, 128.2, 127.7, 127.5, 126.5, 125.1, 67.1, 63.1, 16.7 ppm.

Spectroscopic data are in agreement with the literature.[10]

***trans*-2,3-Bis(4-methylphenyl)oxirane (14)**

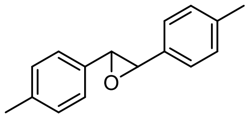

14

Following the general procedure, **pCN-Ur** (10.0 mg), dry MeCN (2.00 mL), isobutyraldehyde (54.8 µL, 0.60 mmol) and 4,4'-dimethyl-*trans*-stilbene (41.7 mg, 0.20 mmol) after 18 h gave a crude mixture containing compound **14** with a yield of 50%, as determined by internal NMR standard. Column chromatography (silica, heptane/EtOAc 99:1) provided the pure product.

^{1}H-NMR (400 MHz, $CDCl_3$): δ = 7.25-7.22 (m, 4 H), 7.20-7.17 (m, 4 H), 3.82 (s, 2 H), 2.37 (s, 6 H) ppm.

^{13}C-NMR (100 MHz, $CDCl_3$): δ = 138.1, 134.2, 129.2, 125.4, 62.8, 21.2 ppm.

Spectroscopic data are in agreement with the literature.[11]

***trans*-2,3-Bis(4-*tert*-butylphenyl)oxirane (15)**

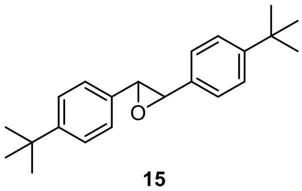

15

Following the general procedure, **pCN-Ur** (10.0 mg), dry MeCN (2.00 mL), isobutyraldehyde (54.8 µL, 0.60 mmol) and 4,4'-di-*tert*-butyl-*trans*-stilbene (58.5 mg, 0.20 mmol) after 18 h gave

a crude mixture containing compound **15** with a yield of 65%, as determined by internal NMR standard. Column chromatography (silica, heptane/EtOAc 99:1) provided the pure product.

^{1}H-NMR (500 MHz, $CDCl_3$): δ = 7.43-7.39 (m, 4 H), 7.30-7.27 (m, 4 H), 3.86 (s, 2 H), 1.34 (s, 18 H) ppm.

^{13}C-NMR (125 MHz, $CDCl_3$): δ = 151.4, 134.3, 125.5, 125.2, 62.7, 34.6, 31.3 ppm.

Spectroscopic data are in agreement with the literature.[11]

trans-2,3-Bis(4-chlorophenyl)oxirane (16)

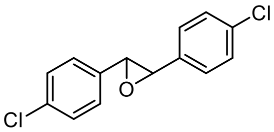

16

Following the general procedure, **pCN-Ur** (10.0 mg), dry MeCN (2.00 mL), isobutyraldehyde (54.8 µL, 0.60 mmol) and 4,4'-dichloro-*trans*-stilbene (49.8 mg, 0.20 mmol) after 18 h gave a crude mixture containing compound **16** with a yield of 55%, as determined by internal NMR standard. Column chromatography (silica, heptane/EtOAc 99:1) provided the pure product.

^{1}H-NMR (500 MHz, $CDCl_3$): δ = 7.38-7.34 (m, 4 H), 7.29-7.25 (m, 4 H), 3.80 (s, 2 H) ppm.

^{13}C-NMR (126 MHz, $CDCl_3$): δ = 135.3, 134.3, 128.8, 126.8, 62.2 ppm.

Spectroscopic data are in agreement with the literature.[11]

trans-2,3-Bis(4-bromophenyl)oxirane (17)

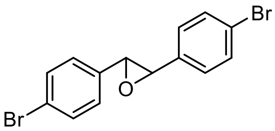

17

Following the general procedure, **pCN-Ur** (10.0 mg), dry MeCN (2.00 mL), isobutyraldehyde (54.8 µL, 0.60 mmol) and 4,4'-dibromo-*trans*-stilbene (67.6 mg, 0.20 mmol) after 18 h gave a crude mixture containing compound **18** with a yield of 46%, as determined by internal NMR standard. Column chromatography (silica, heptane/EtOAc 99:1) provided the pure product.

^{1}H-NMR (400 MHz, $CDCl_3$): δ = 7.52-7.50 (m, 4 H), 7.22-7.18 (m, 4 H), 3.78 (s, 2 H) ppm.

^{13}C-NMR (100 MHz, $CDCl_3$): δ = 135.8, 131.8, 127.1, 122.4, 62.2 ppm.

Spectroscopic data are in agreement with the literature.[12]

6.2 Synthesis of Rose oxide including chromatographic purification of intermediates

Preparation of epoxide **4** on 2 mmol scale:

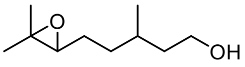

4

An oven-dried 100 mL reaction tube (diameter 3 cm) was charged with photocatalyst **pCN-Ur** (100 mg), dry MeCN (20 mL), isobutyraldehyde (548 µL, 6.00 mmol) and (±)-citronellol (**1**, 363 µL, 2.00 mmol). After sealing the tube with a septum, O_2 was bubbled through the solution via cannula for 15 min and an O_2-filled balloon was attached to provide an O_2 atmosphere of 1 bar during the reaction. The reaction mixture was stirred and irradiated for 15.5 h (blue LED, *hv* = 450 ± 25 nm, 18 W, 34 mW·cm^{-2}, EvoluChem^{TM} PhotoRedOx Box, internal temperature 30 °C). After filtration through a fritted glass funnel, the reaction mixture was washed with aq. 1 M $Na_2S_2O_3$ solution and extracted with Et_2O (3×). The combined organic layers were dried over Na_2SO_4, filtered, and the solvent was removed under reduced pressure. Column chromatography over deactivated silica gel (heptane/EtOAc 2:1 → 1:1, silica pre-treated with 2 % Et_3N solution) provided epoxide **4** (colorless oil, 280.2 mg, 81%).

Preparation of diols **18a** and **18b**:

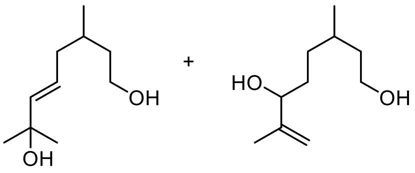

18a + 18b

In an oven-dried 25 mL round-bottom flask citronellol epoxide **4** (280.2 mg, 1.63 mmol) was dissolved in dry DMSO (9.00 mL) under argon. Potassium *tert*-butoxide (365.8 mg, 3.26 mmol, 2 eq.) was added. The mixture was heated to 60 °C for 1 h, resulting in an orange-brown color. 10 mL of H_2O were added, and the mixture was extracted with Et_2O (3×). The organic phase was washed with a small amount of H_2O, dried with Na_2SO_4, filtered, and the solvent was removed under reduced pressure. Column chromatography over deactivated silica gel (heptane/EtOAc 1:1 → 1:2, silica pre-treated with 2 % Et_3N solution) provided the mixture of diols **18a** and **18b** (colorless oil, 241.6 mg, 86 %). The ratio of diols was determined by ^{1}H-NMR analysis (**18a**:**18b** = 3.1:1). Further chromatographic purifications provided analytical samples of the individual diols in pure form (*d.r.* of **18 b** ca. 2:1).

Compound **18a**:

^{1}H-NMR (500 MHz, $CDCl_3$): δ = 5.63-5.55 (m, 2 H), 3.72-3.61 (m, 2 H), 2.06-2.01 (m, 1 H), 1.93-1.88 (m, 1 H), 1.70-1.55 (m, 3 H), 1.43-1.33 (m, 1 H), 1,30 (s, 6 H), 0.89 (d, J = 6.7 Hz, 3 H) ppm.

^{13}C-NMR (126 MHz, $CDCl_3$): δ = 139.6, 125.2, 70.7, 61.0, 39.7, 39.3, 29.85, 29.81, 29.7, 19.5 ppm.

Spectroscopic data are in agreement with the literature.[13]

Compound **18b** (*d.r.* ca. 1.5:1):

^{1}H-NMR (500 MHz, $CDCl_3$): δ = 4.94-4.93 (m, 1 H), 4.85-4.83 (m, 1 H), 4.06-4.03 (m, 1 H), 3.74-3.64 (m, 2 H), 1.72 (s, 3 H), 1.66-1.21 (m), 1.16-1.06 (m, 1 H), 0.93-0.90 (m, 3 H) ppm.

^{13}C-NMR (126 MHz, $CDCl_3$): δ = 147.6, 147.5, 111.2, 111.0, 76.3, 76.1, 61.1, 39.8, 39.7, 32.7, 32.6, 32.1, 29.5, 29.3, 19.64, 19.62, 17.6, 17.4 ppm.

Spectroscopic data are in agreement with the literature.[13]

Preparation of (±)-Rose oxide (**19**):

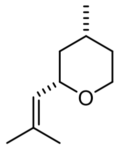

(±)-Rose oxide (**19**)

An oven-dried 25 mL two-necked flask was equipped with a reflux condenser, evacuated and backfilled with argon. After charging with a mixture of 30 wt.-% aqueous H_2SO_4 (2.70 mL) and Et_2O (1.30 mL) the solution was heated to 30 °C and stirred vigorously. The mixture of diols **18a** and **18b** (241.6 mg, 1.40 mmol) was dissolved in 0.50 mL of Et_2O and added dropwise to the mixture over 30 min. After stirring for 30 more minutes, the solution was cooled to r.t. for 1 h. The reaction mixture was transferred into a separation funnel and the layers were separated, and the aqueous phase was extracted Et_2O (3×). The combined organic layers were washed with 5 wt.-% aq. Na_2CO_3 solution and the solvent was carefully removed under reduced pressure. The pure product was isolated by short path distillation (120 °C, 20 mbar) and the *cis*:*trans* ratio was determined by ^{1}H-NMR analysis (colorless oil, 116.3 mg, 71 % with respect to compound **18a**, *cis*:*trans* = 2.5:1).

cis/trans-mixture:

^{1}H-NMR (400 MHz, $CDCl_3$): δ = 5.29-5.25 (m, 1 H), 5.16-5.12 (m, 2.5 H), 4.34 (td, J = 8.0, 3.1 Hz, 1 H), 4.01-3.93 (m, 5 H), 3.75-3.64 (m, 2 H), 3.48-3.41 (m, 2.5 H), 2.03-1.94 (m, 1 H), 1.86-.165 (m, 24 H), 1.64-1.47 (m, 9 H), 1.37-1.31 (m, 1 H), 1.14-1.26 (m, 4 H), 1.05 (d, J = 7.1 Hz, 3 H), 1.03-0.97 (m, 2 H), 0.92 (d, J = 6.5 Hz, 7.5 H) ppm.

cis-Isomer:

^{13}C-NMR (100 MHz, $CDCl_3$): δ = 135.0, 126.3, 74.6, 67.8, 40.8, 34.4, 30.2, 25.6, 22.3, 18.3 ppm.

trans-Isomer:

^{13}C-NMR (100 MHz, $CDCl_3$): δ = 135.5, 125.3, 69.0, 62.1, 38.1, 32.4, 25.7, 24.9, 19.1, 18.2 ppm.

Spectroscopic data are in agreement with the literature.[14]

6.3 Synthesis of Rose oxide without chromatographic purification of intermediates

Preparation of epoxide **4** on 2 mmol scale:

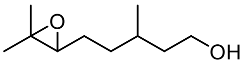

4

An oven-dried 100 mL reaction tube (diameter 3 cm) was charged with photocatalyst **pCN-Ur** (100 mg), dry MeCN (20 mL), isobutyraldehyde (548 µL, 6.00 mmol) and (±)-citronellol (**1**, 363 µL, 2.00 mmol). After sealing the tube with a septum, O_2 was bubbled through the solution via cannula for 15 min and an O_2-filled balloon was attached to provide an O_2 atmosphere of 1 bar during the reaction. The reaction mixture was stirred and irradiated for 17 h (blue LED, *hv* = 450 ± 25 nm, 18 W, 34 mW·cm^{-2}, EvoluChem^{TM} PhotoRedOx Box, internal temperature 30 °C). After filtration through a fritted glass funnel, the reaction mixture was washed with aq. 1 M $Na_2S_2O_3$ solution and extracted with Et_2O (3×). The combined organic layers were dried over Na_2SO_4, filtered, and the solvent was removed under reduced pressure. The yield of epoxide **4** was determined by ^{1}H-NMR against CH_2Br_2 standard, to be 82%.

Preparation of diols **18a** and **18b**:

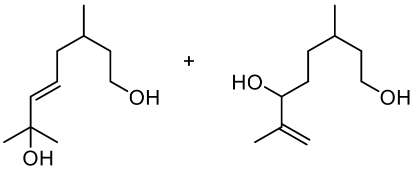

18a + **18b**

In an oven-dried 25 mL round-bottom flask potassium *tert*-butoxide (552.1 mg, 4.92 mmol, 3 eq.) was dissolved in dry DMSO (8.00 mL) under argon and the mixture was heated to 60 °C. The crude citronellol epoxide **4** (1.64 mmol, 1 eq.) was dissolved in 1.00 mL of dry DMSO and added to the reaction mixture dropwise. After stirring at 60 °C for 1 h, the orange-brown mixture was cooled to room temperature. 10 mL of H_2O were added, and the product was extracted with Et_2O (3×). The organic phase was washed with a small amount of H_2O, dried with Na_2SO_4, filtered, and the solvent was removed under reduced pressure. The yield of diols **18a** and **18b** was determined by ^{1}H-NMR against CH_2Br_2 standard, to be 74% (ratio **18a**:**18b** = 2.4:1).

Preparation of (±)-Rose oxide (**19**):

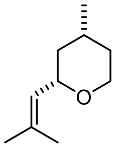

(±)-Rose oxide (**19**)

An oven-dried 25 mL two-necked flask was equipped with a reflux condenser, evacuated and backfilled with argon. After charging with a mixture of 30 wt.-% aqueous H_2SO_4 (4.00 mL) and Et_2O (2.00 mL) the solution was heated to 30 °C and stirred vigorously. The mixture of diols

18a and **18b** (max. 1.22 mmol) was dissolved in 1.00 mL of Et_2O and added dropwise to the mixture over 30 min. After stirring for 30 more minutes, the solution was cooled to r.t. for 1 h. The reaction mixture was transferred into a separation funnel and the layers were separated, and the aqueous phase was extracted Et_2O (3×). The combined organic layers were washed with 5 wt.-% aq. Na_2CO_3 solution and the solvent was carefully removed under reduced pressure. The pure product was isolated by short path distillation (120 °C, 20 mbar) and the *cis*:*trans* ratio was determined by ^{1}H-NMR analysis (colorless oil, 96.6 mg, 31 % over 3 steps from citronellol **1**, *cis*:*trans* = 5:1).

7 NMR Spectra

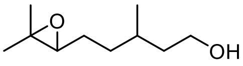

4 *d.r.* 1:1

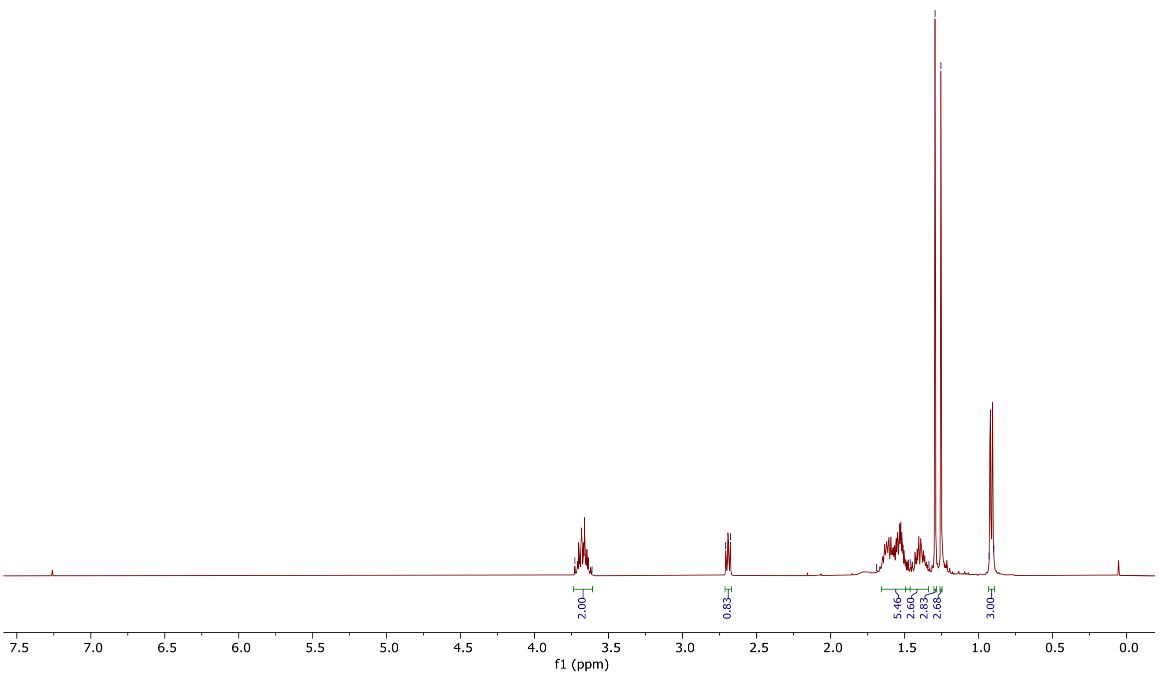

^{1}H-NMR (400 MHz, $CDCl_3$)

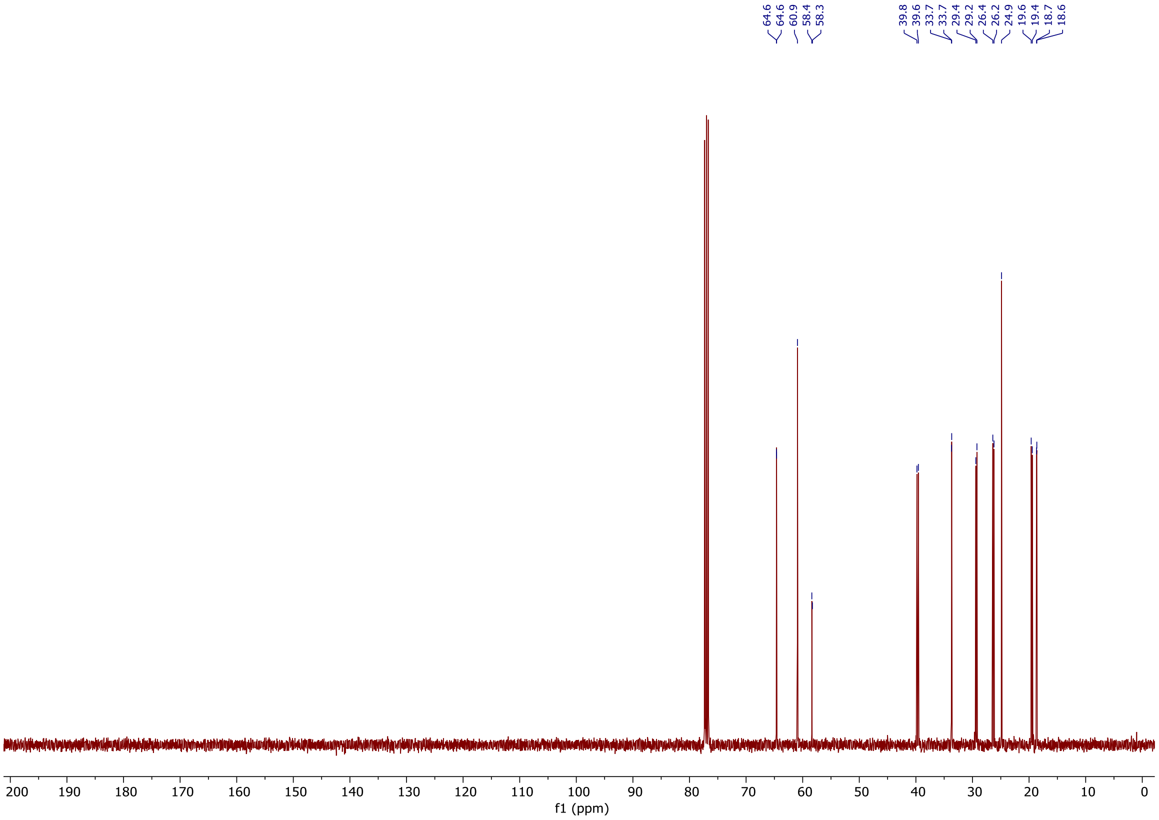

^{13}C-NMR (100 MHz, $CDCl_3$)

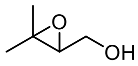

5

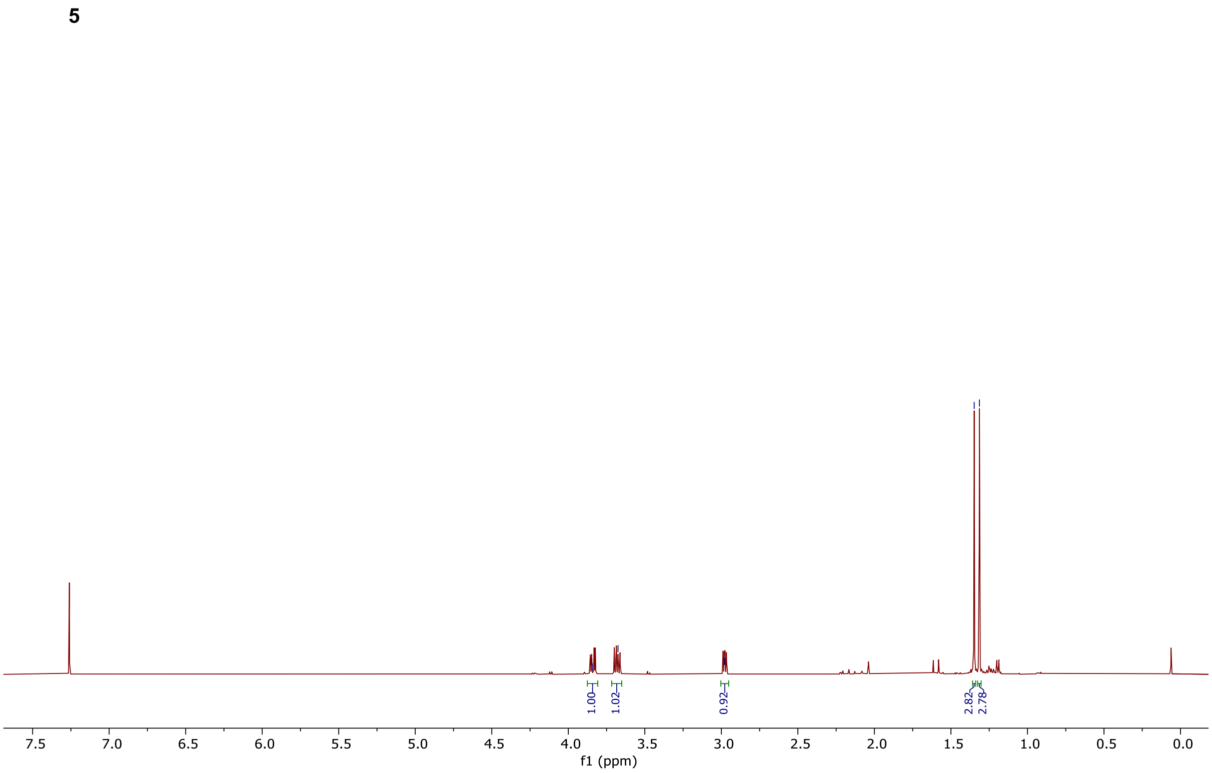

^{1}H-NMR (500 MHz, $CDCl_3$)

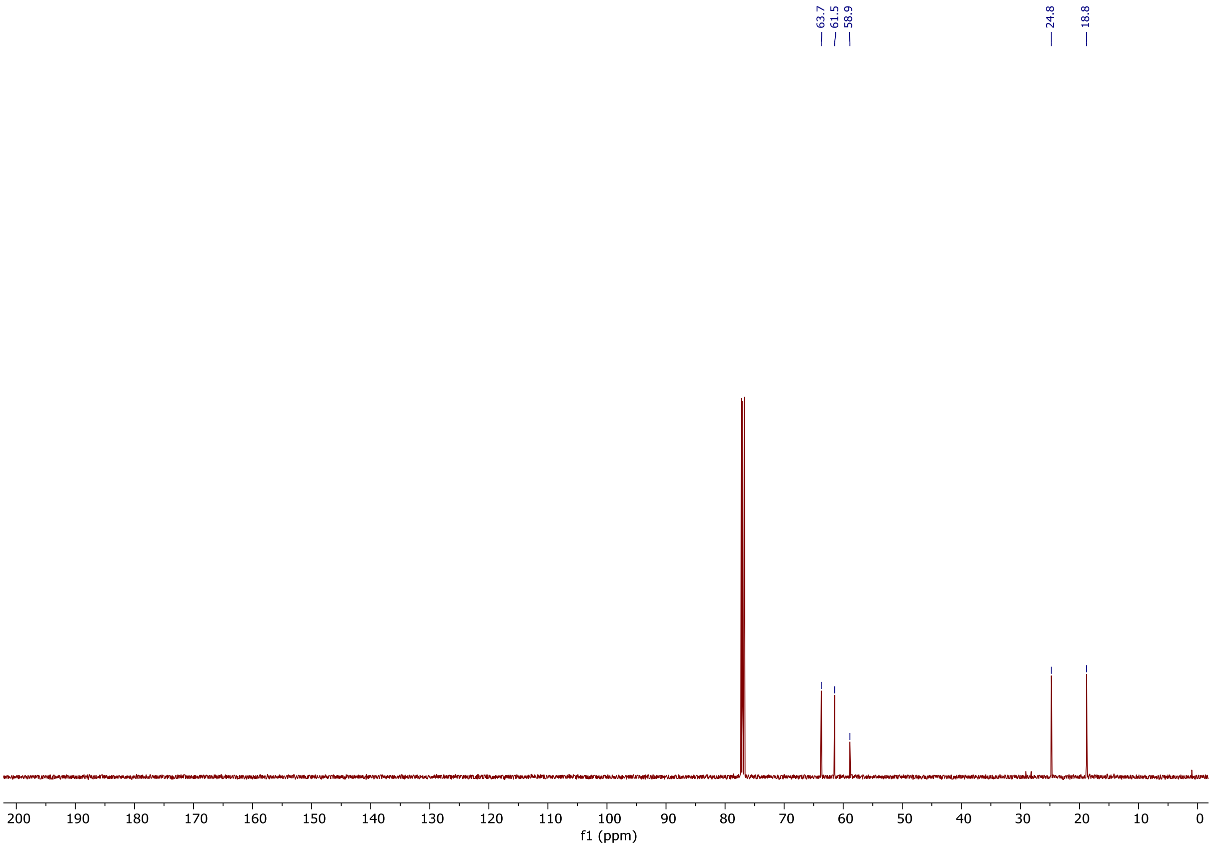

^{13}C-NMR (125 MHz, $CDCl_3$)

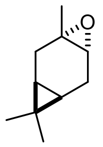

6

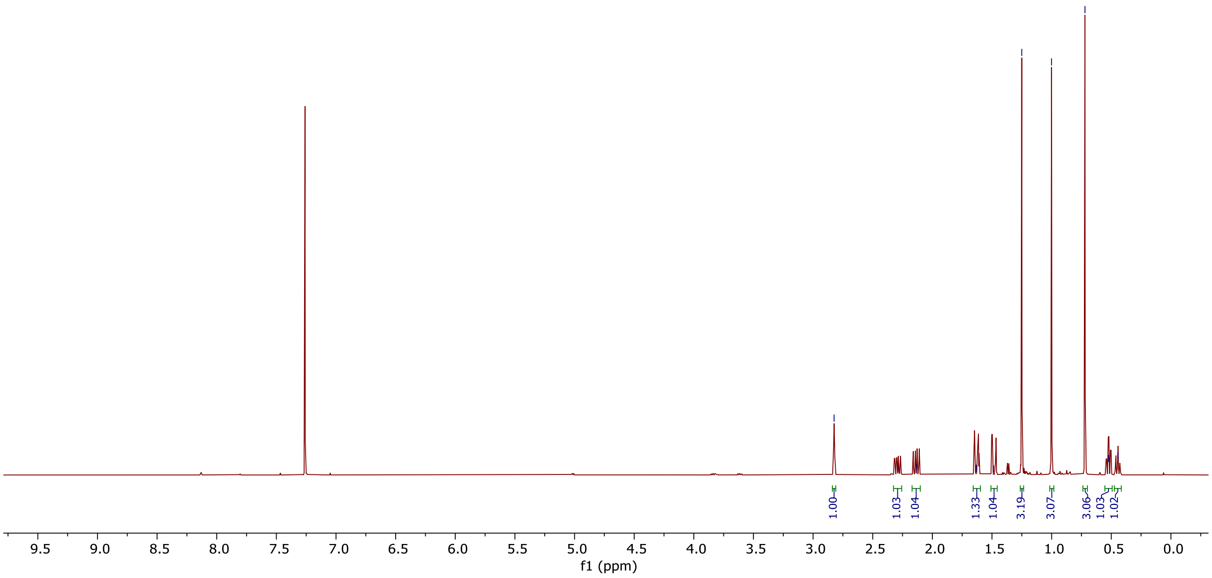

¹H-NMR (500 MHz, $CDCl_3$)

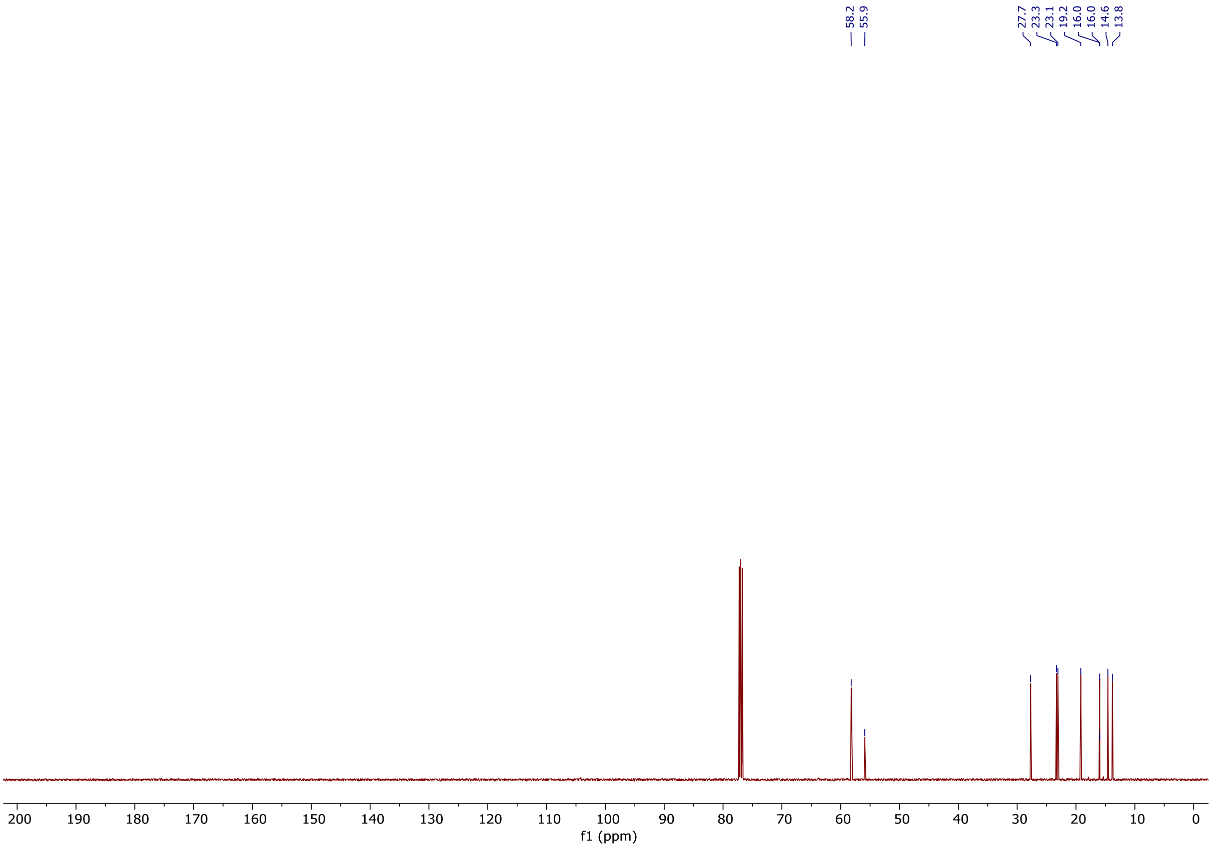

¹³C-NMR (125 MHz, $CDCl_3$)

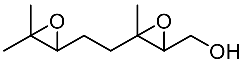

8

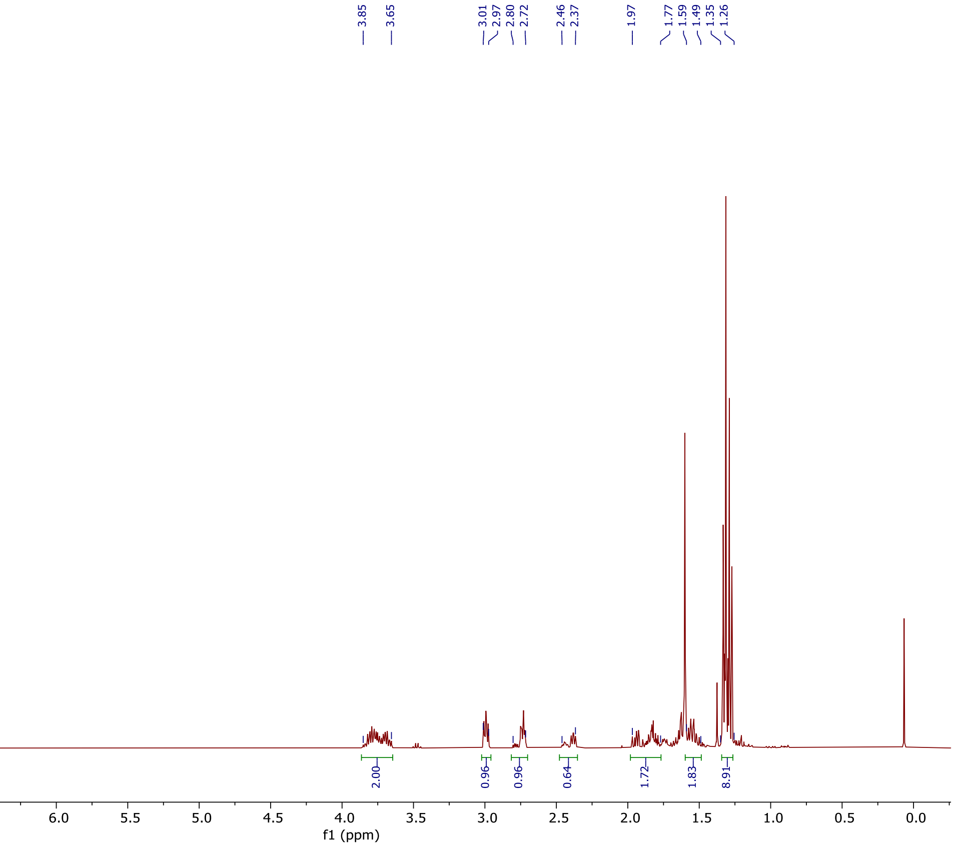

¹H-NMR (400 MHz, $CDCl_3$)

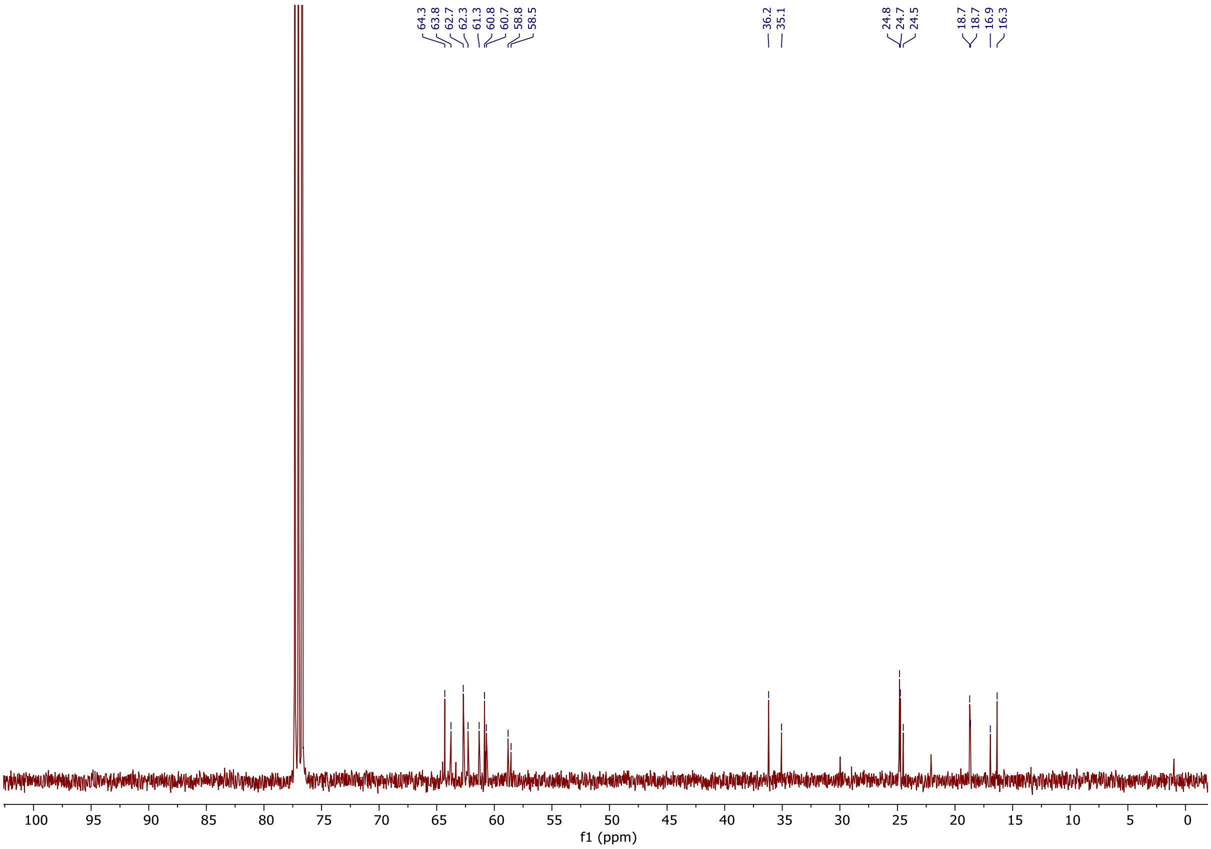

¹³C-NMR (100 MHz, $CDCl_3$)

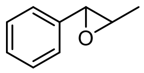

9

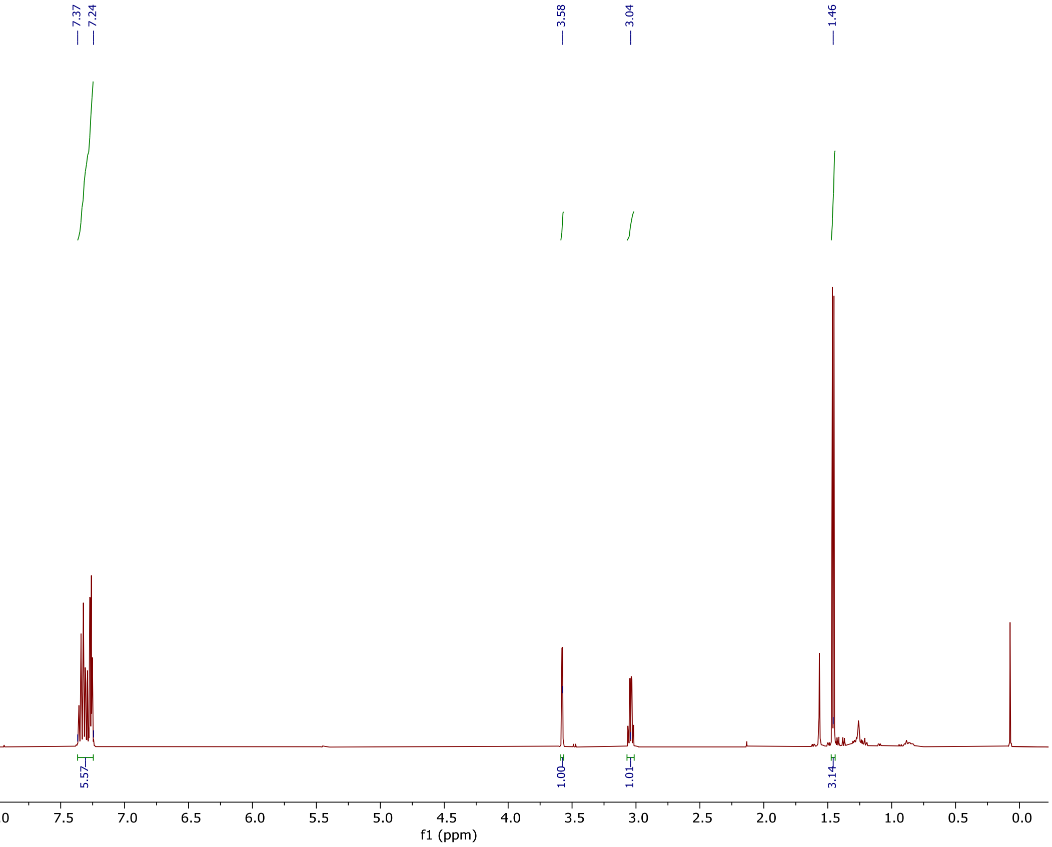

^{1}H-NMR (400 MHz, $CDCl_3$)

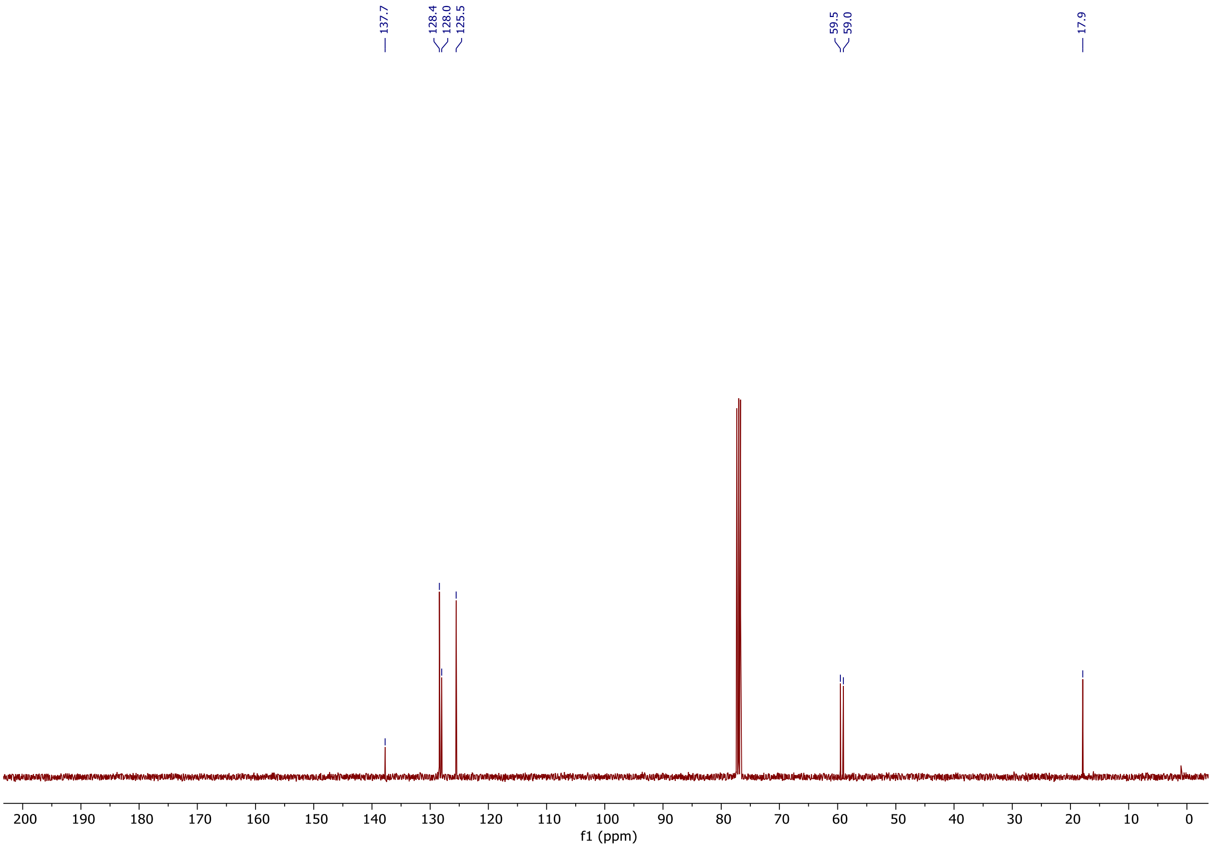

^{13}C-NMR (100 MHz, $CDCl_3$)

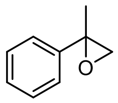

10

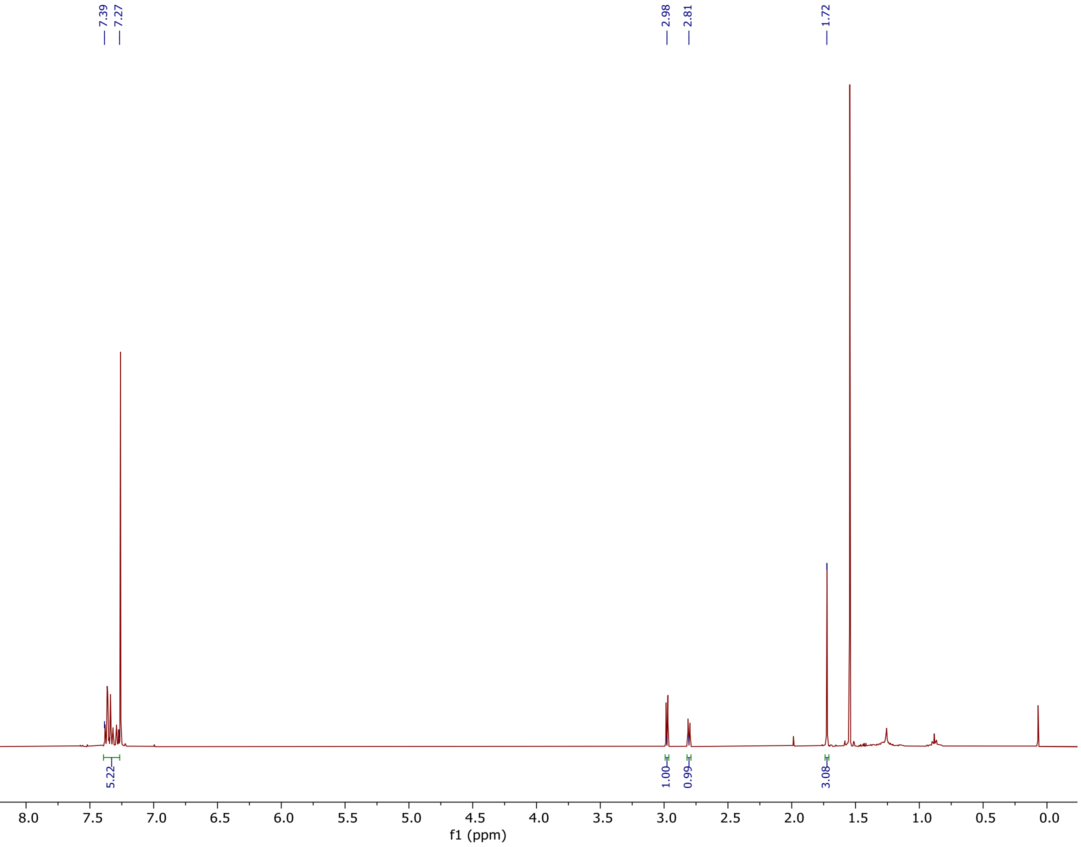

^{1}H-NMR (400 MHz, $CDCl_3$)

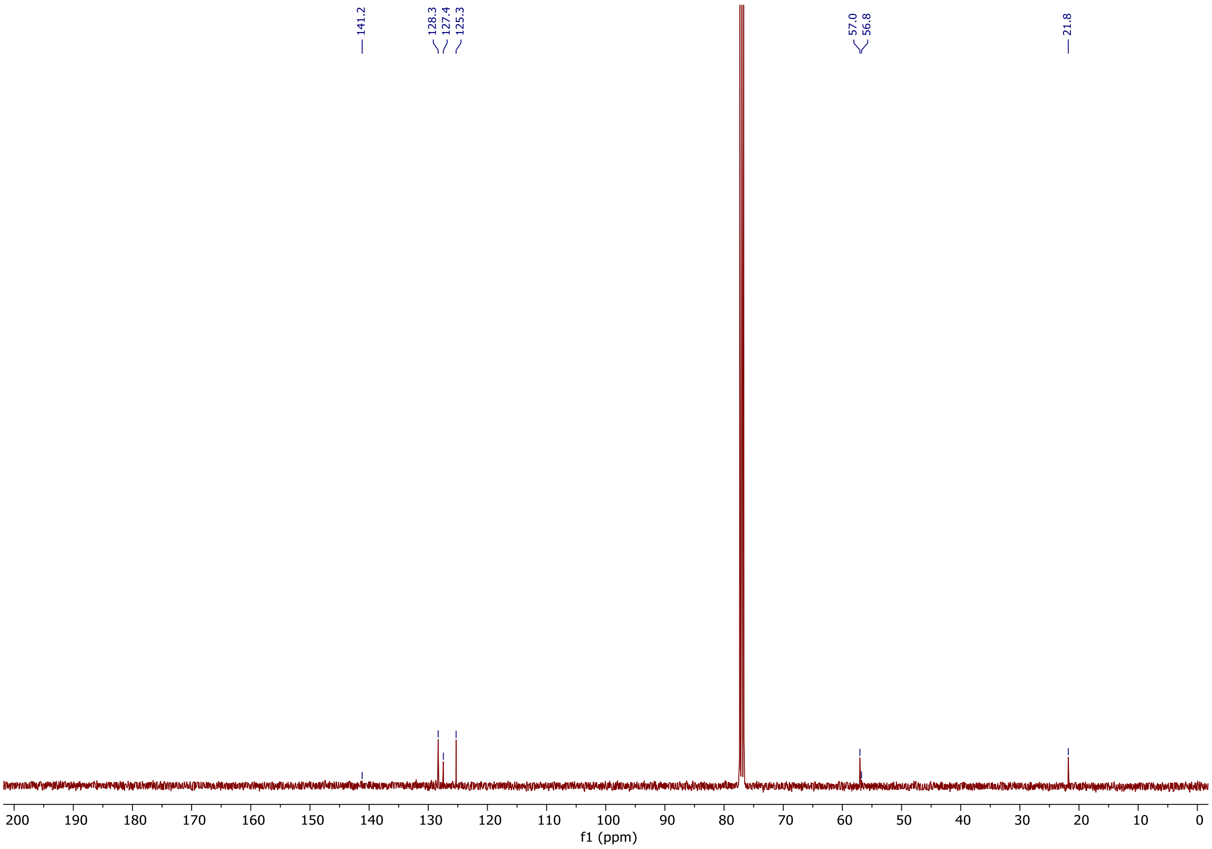

^{13}C-NMR (100 MHz, $CDCl_3$)

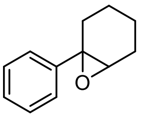

11

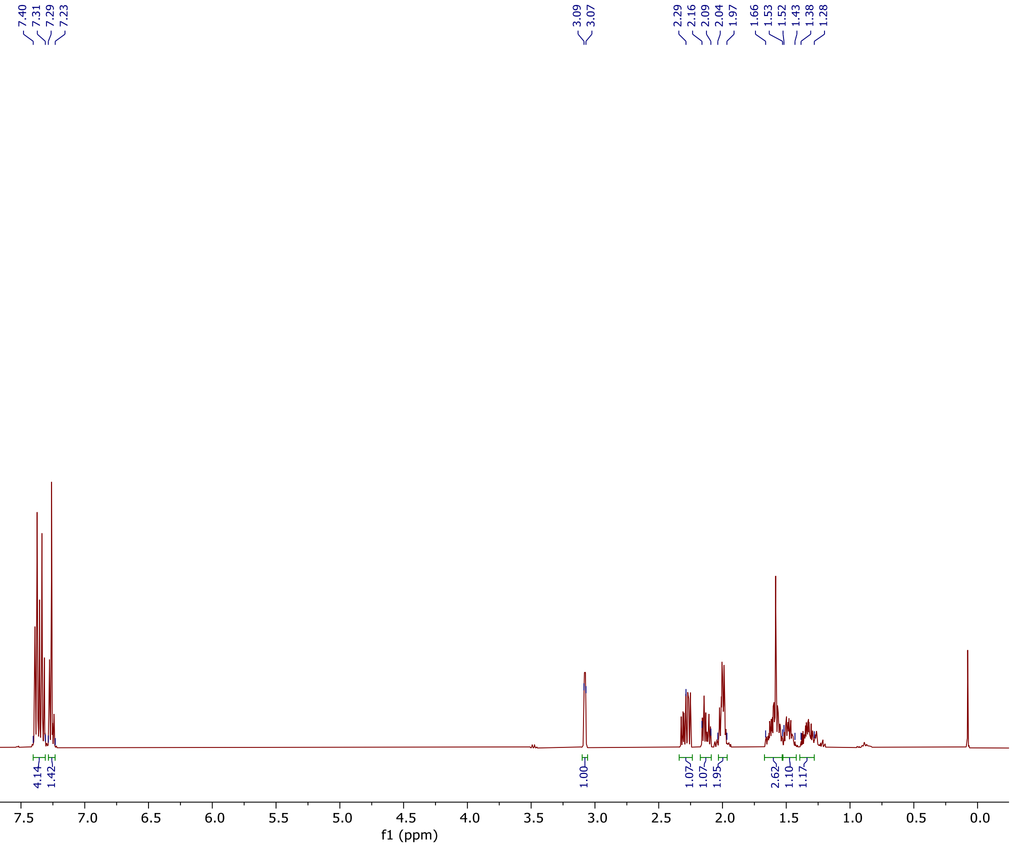

¹H-NMR (400 MHz, $CDCl_3$)

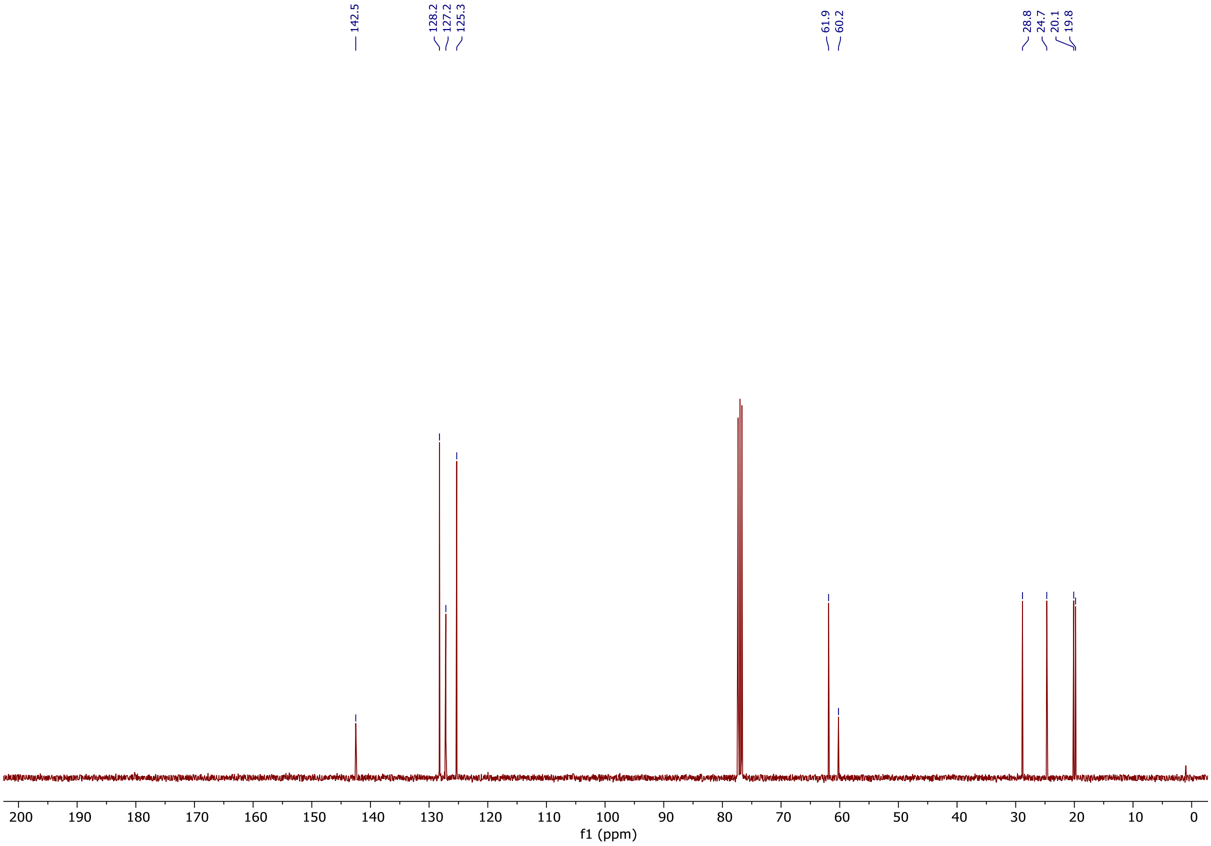

¹³C-NMR (100 MHz, $CDCl_3$)

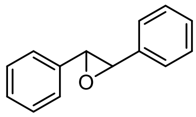

12

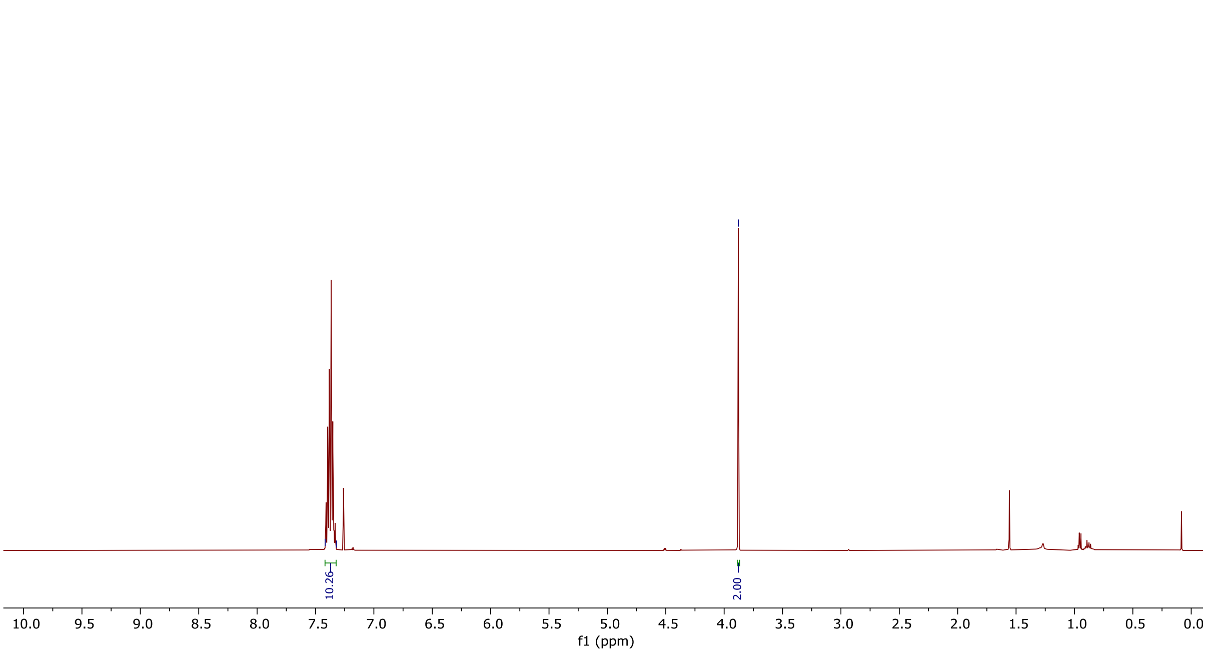

^{1}H-NMR (500 MHz, $CDCl_3$)

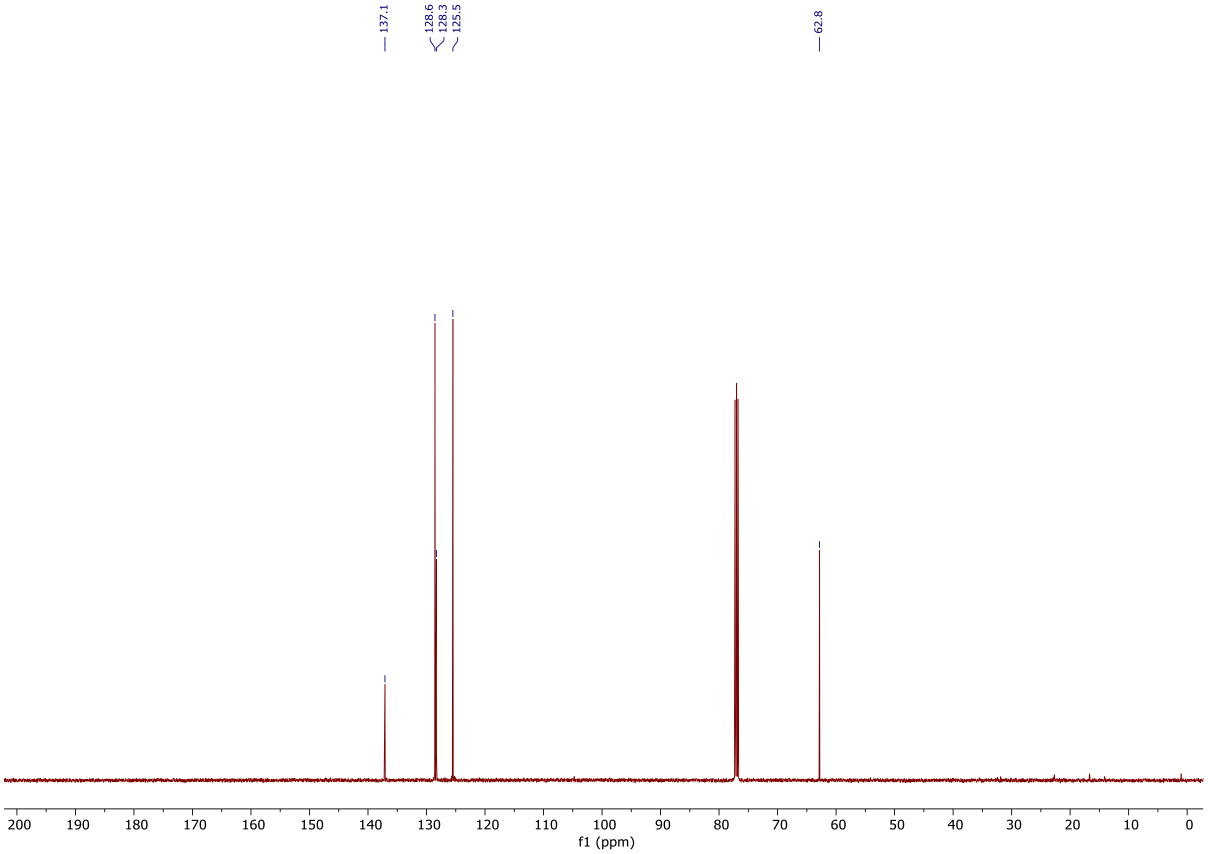

^{13}C-NMR (126 MHz, $CDCl_3$)

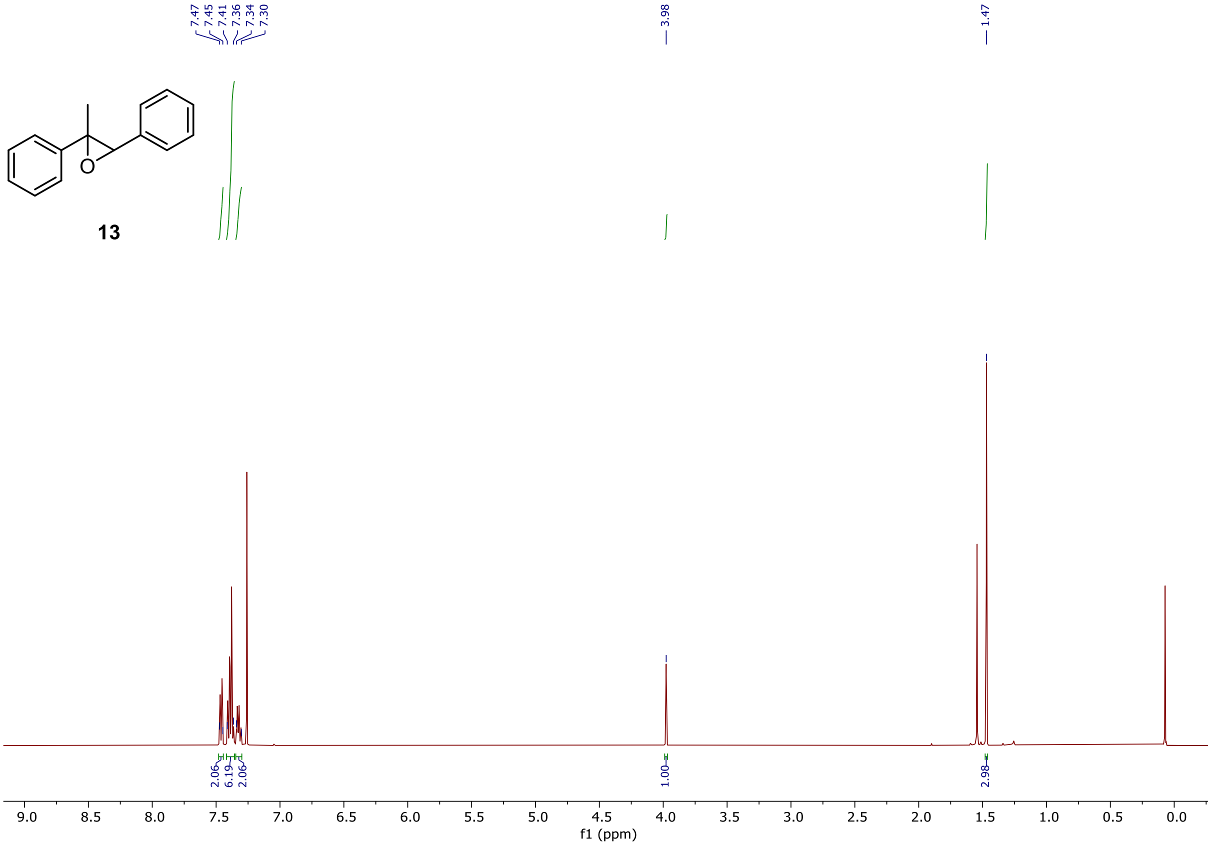

13

^{1}H-NMR (500 MHz, $CDCl_3$)

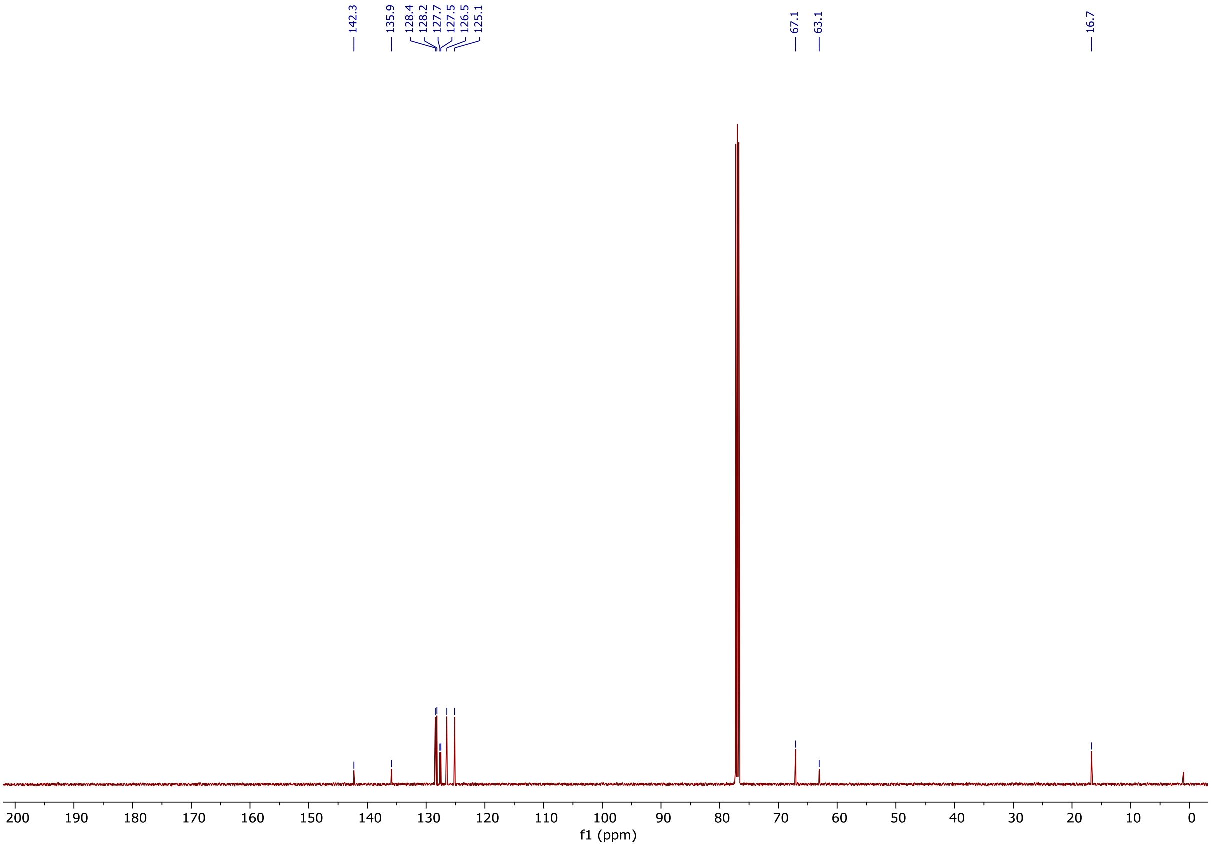

^{13}C-NMR (126 MHz, $CDCl_3$)

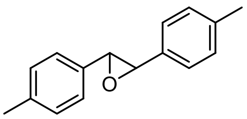

14

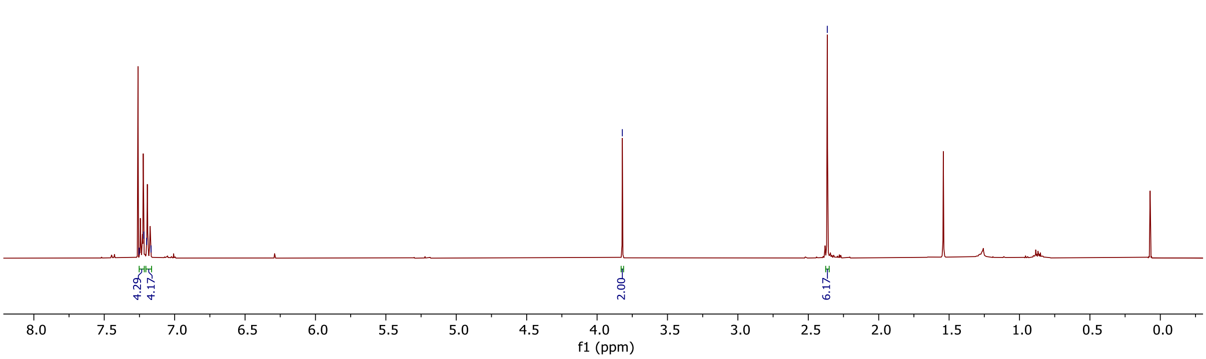

^{1}H-NMR (400 MHz, $CDCl_3$)

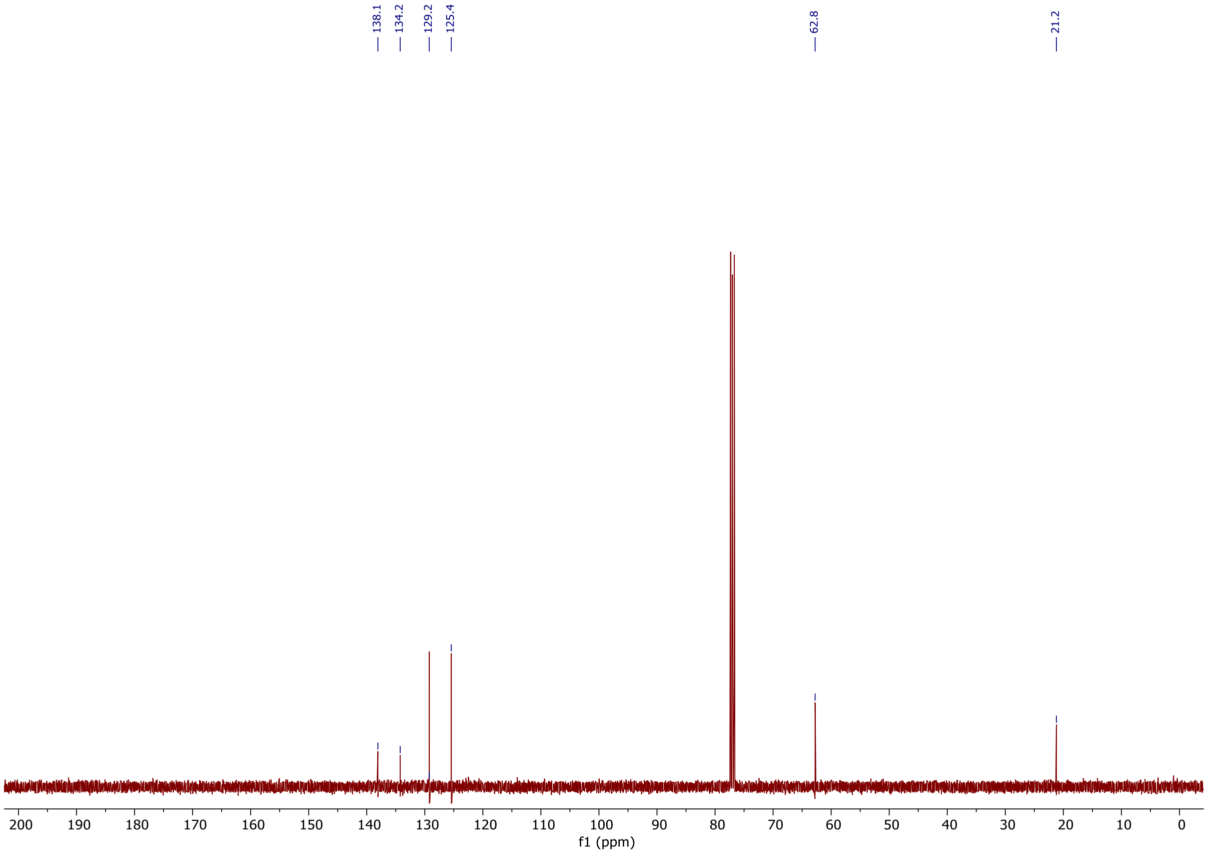

^{13}C-NMR (100 MHz, $CDCl_3$)

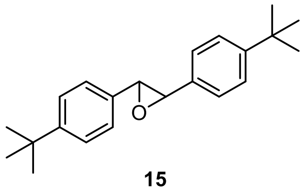

15

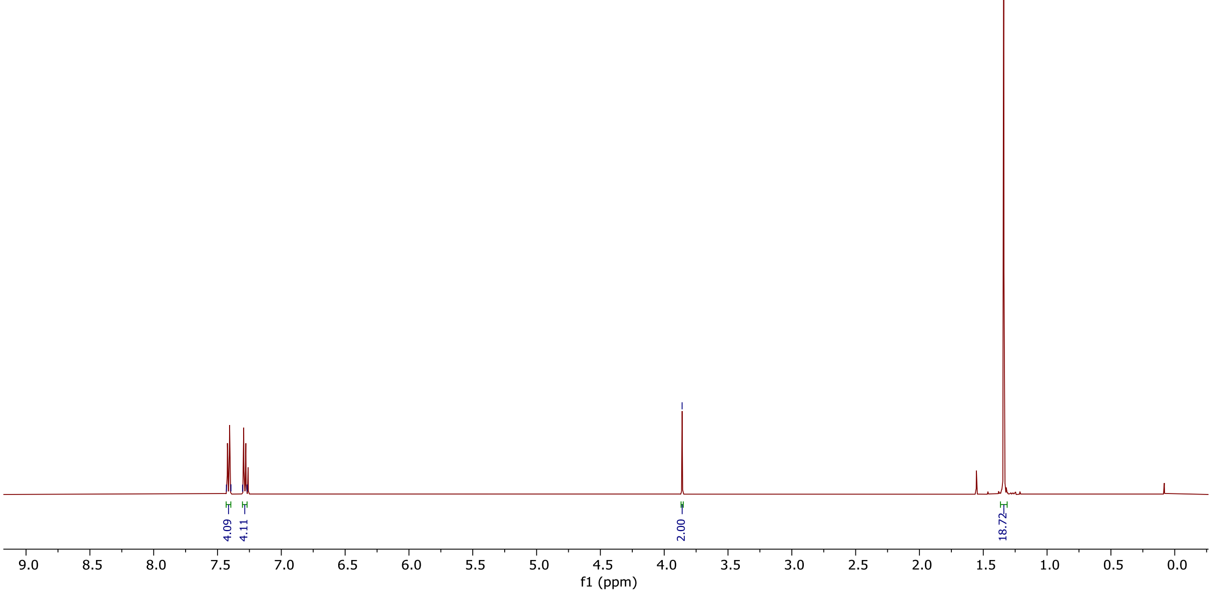

^{1}H-NMR (500 MHz, $CDCl_3$)

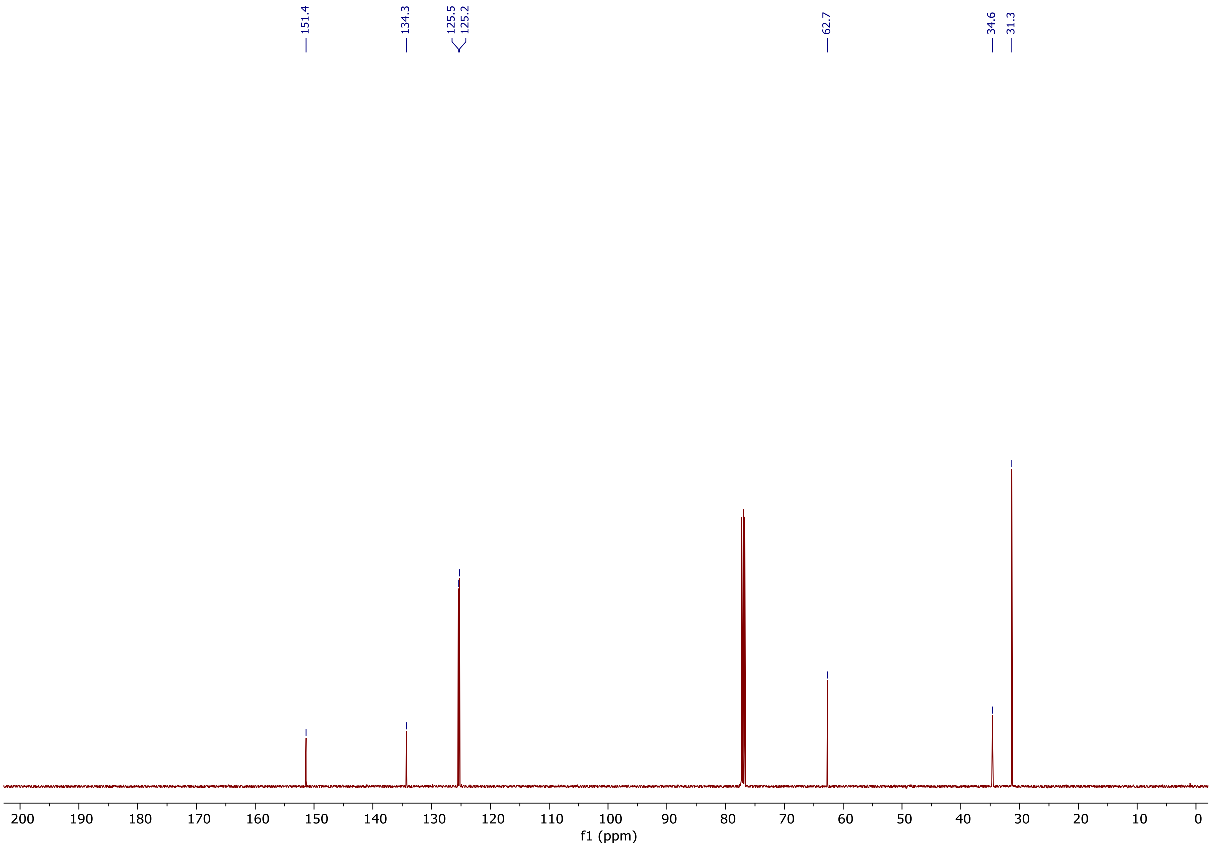

^{13}C-NMR (126 MHz, $CDCl_3$)

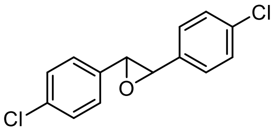

16

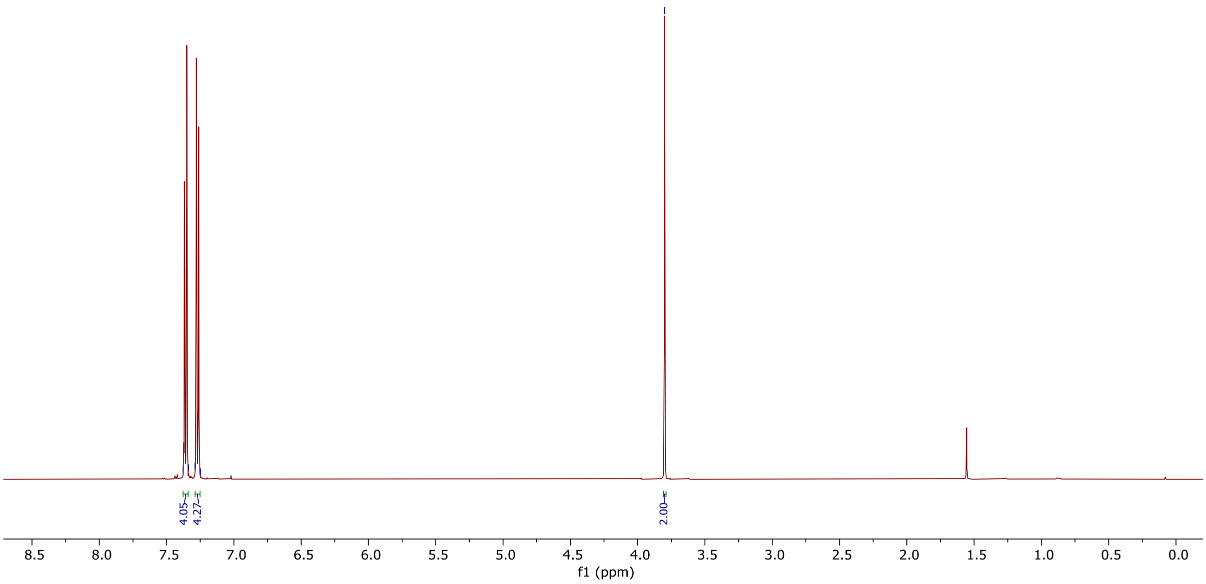

^{1}H-NMR (500 MHz, $CDCl_3$)

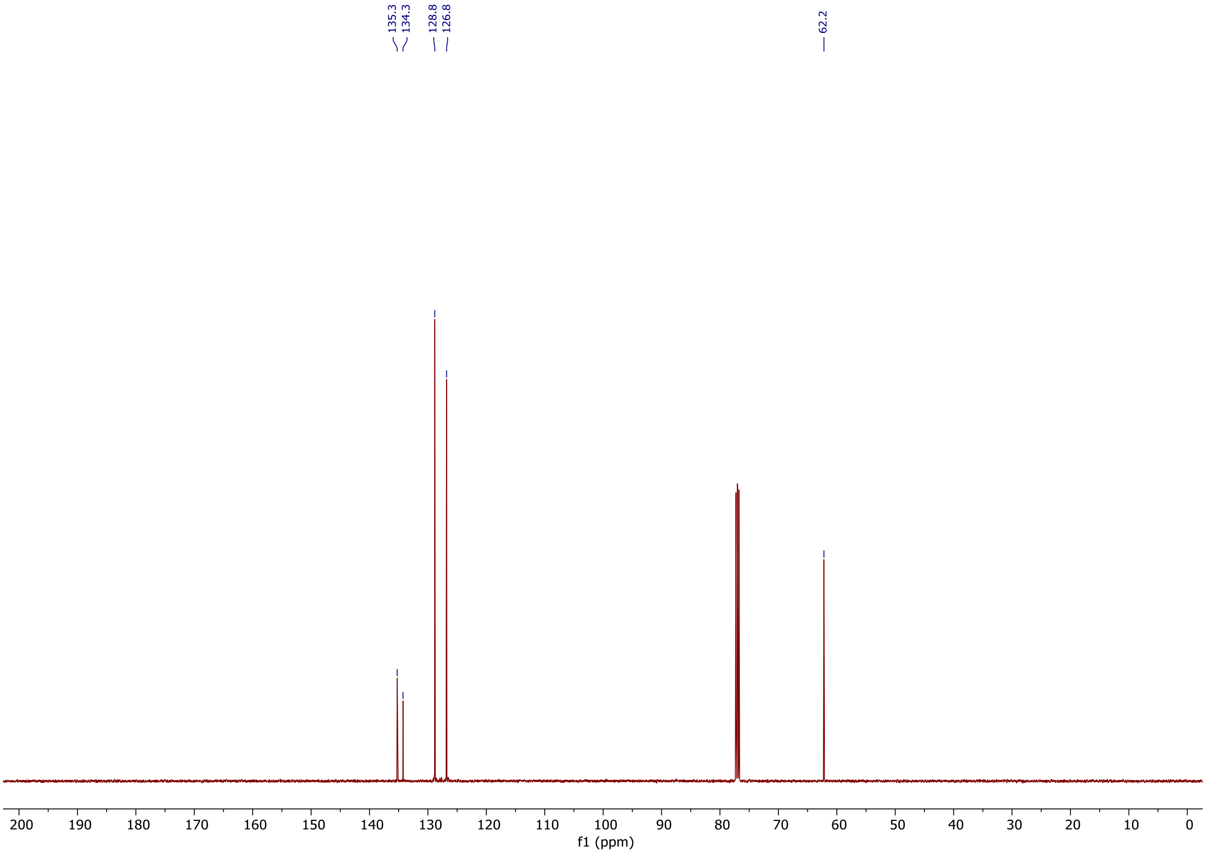

^{13}C-NMR (126 MHz, $CDCl_3$)

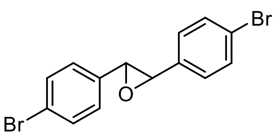

17

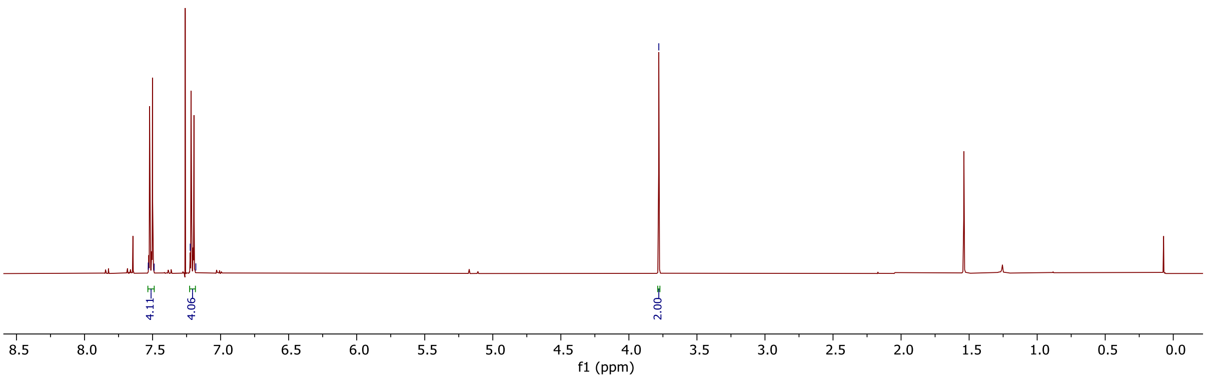

^{1}H-NMR (400 MHz, $CDCl_3$)

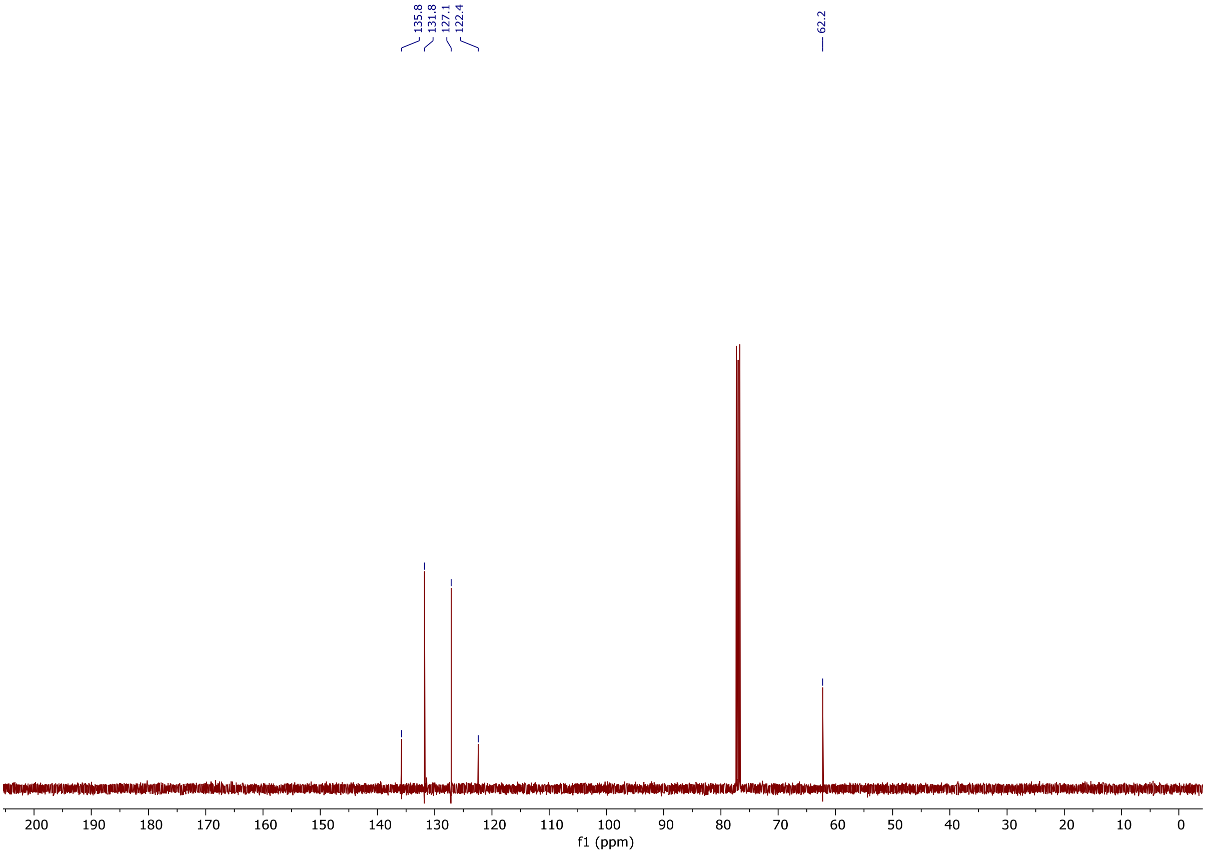

^{13}C-NMR (100 MHz, $CDCl_3$)

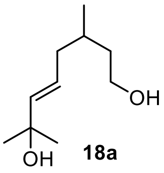

18a

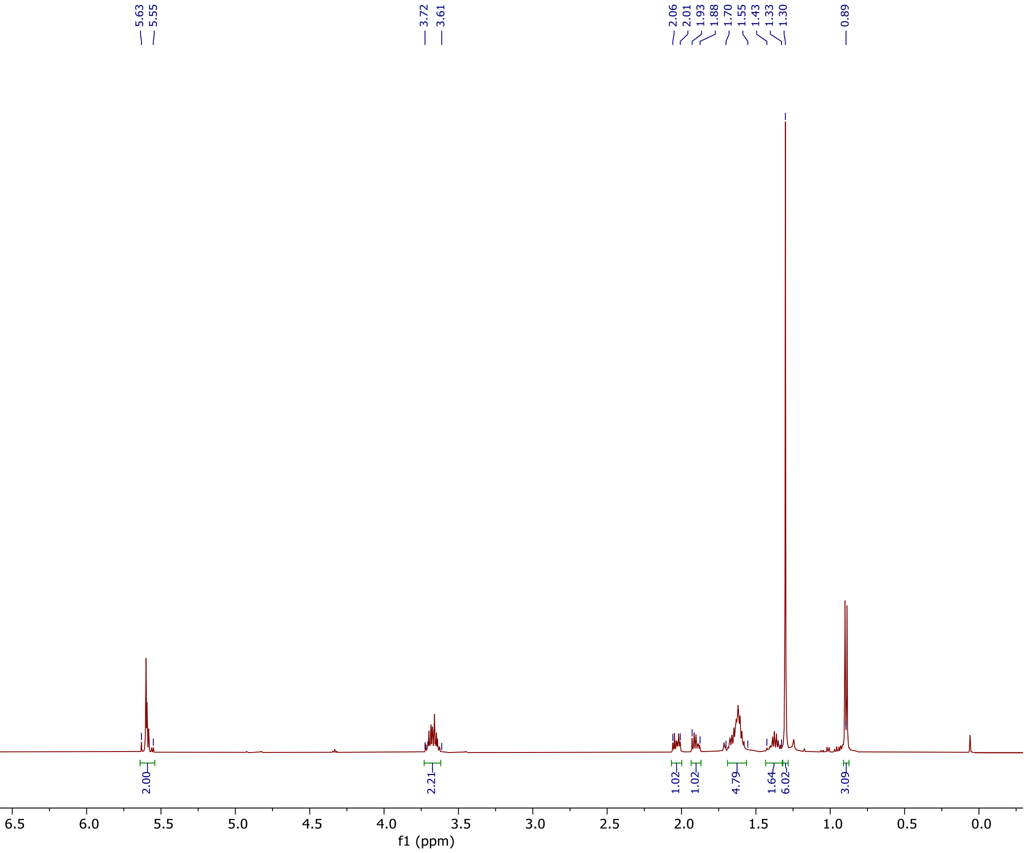

^{1}H-NMR (500 MHz, $CDCl_3$)

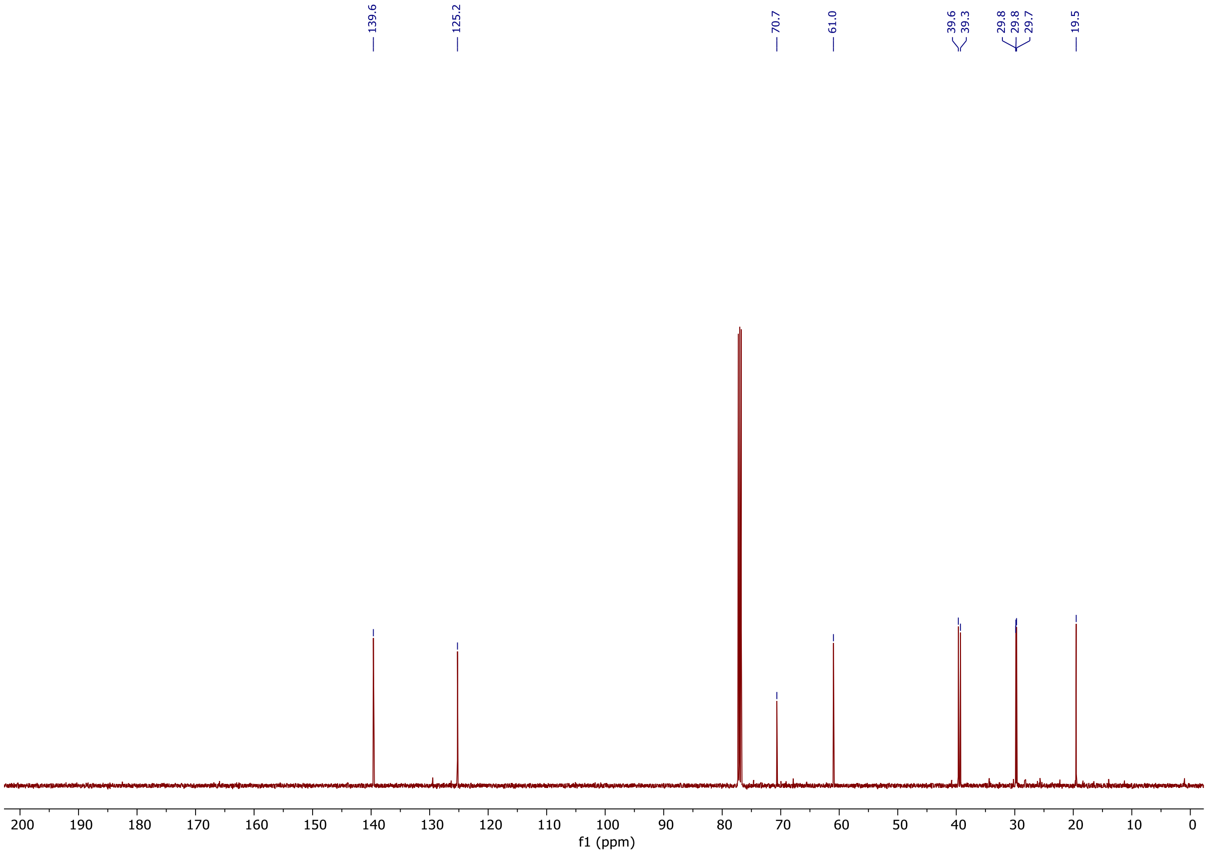

^{13}C-NMR (126 MHz, $CDCl_3$)

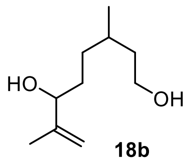

18b

d.r. 1.5:1

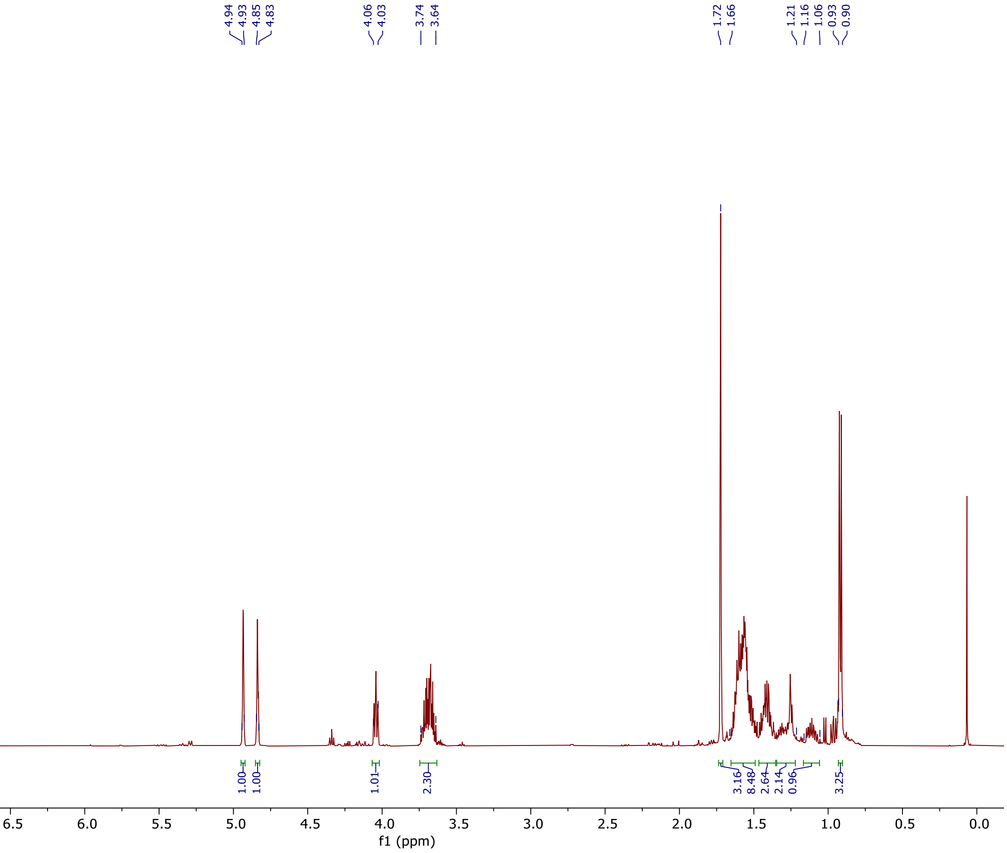

^{1}H-NMR (500 MHz, $CDCl_3$)

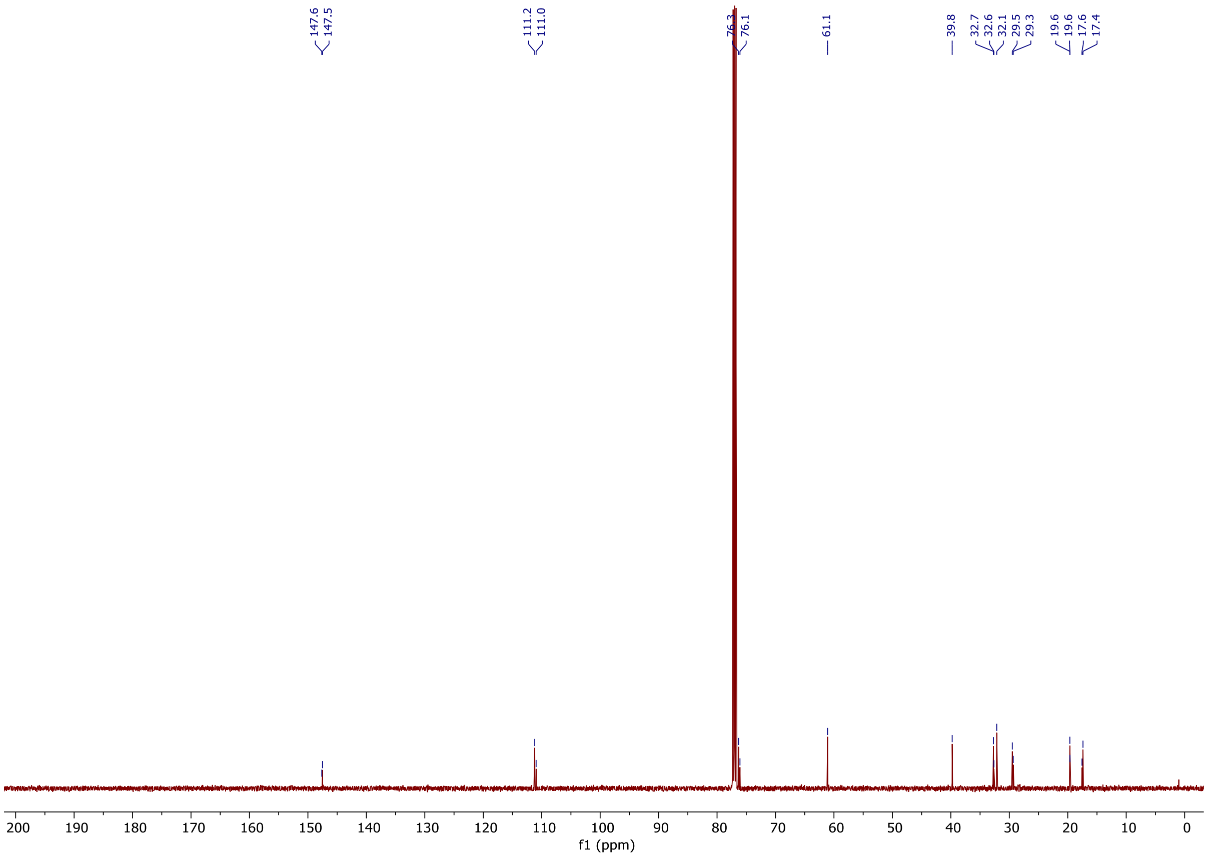

^{13}C-NMR (126 MHz, $CDCl_3$)

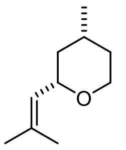

(±)-Rose oxide (**19**)
d.r. 2.5:1

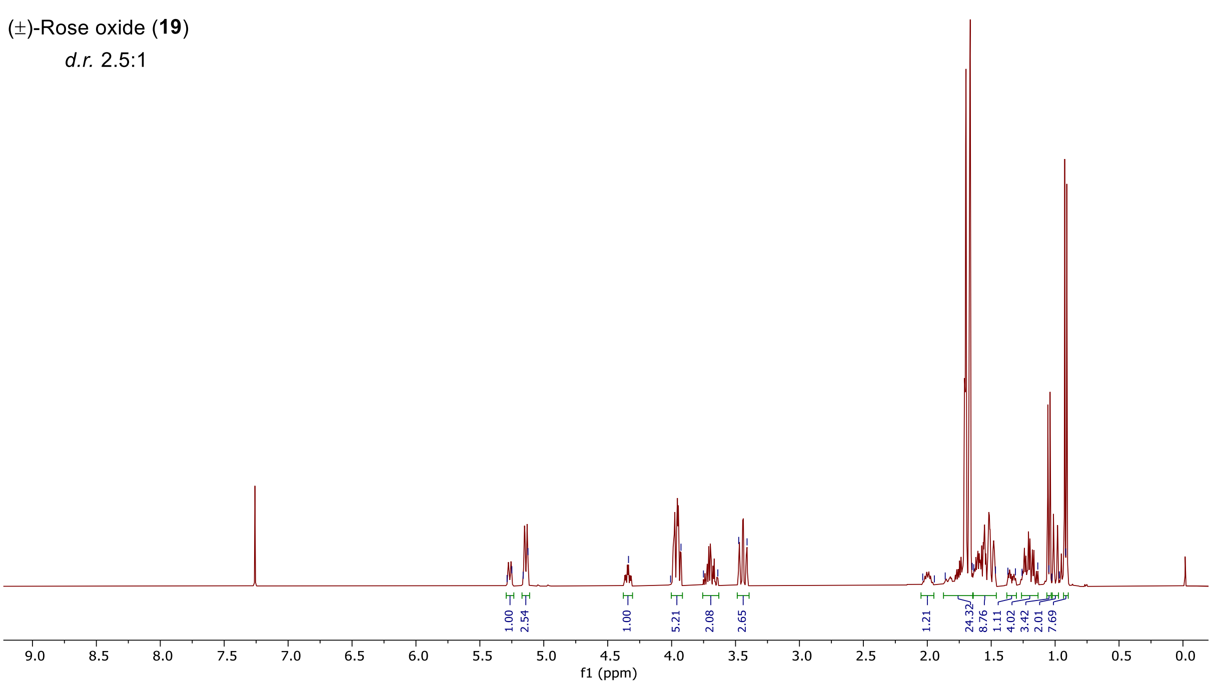

^{1}H-NMR (400 MHz, $CDCl_3$)

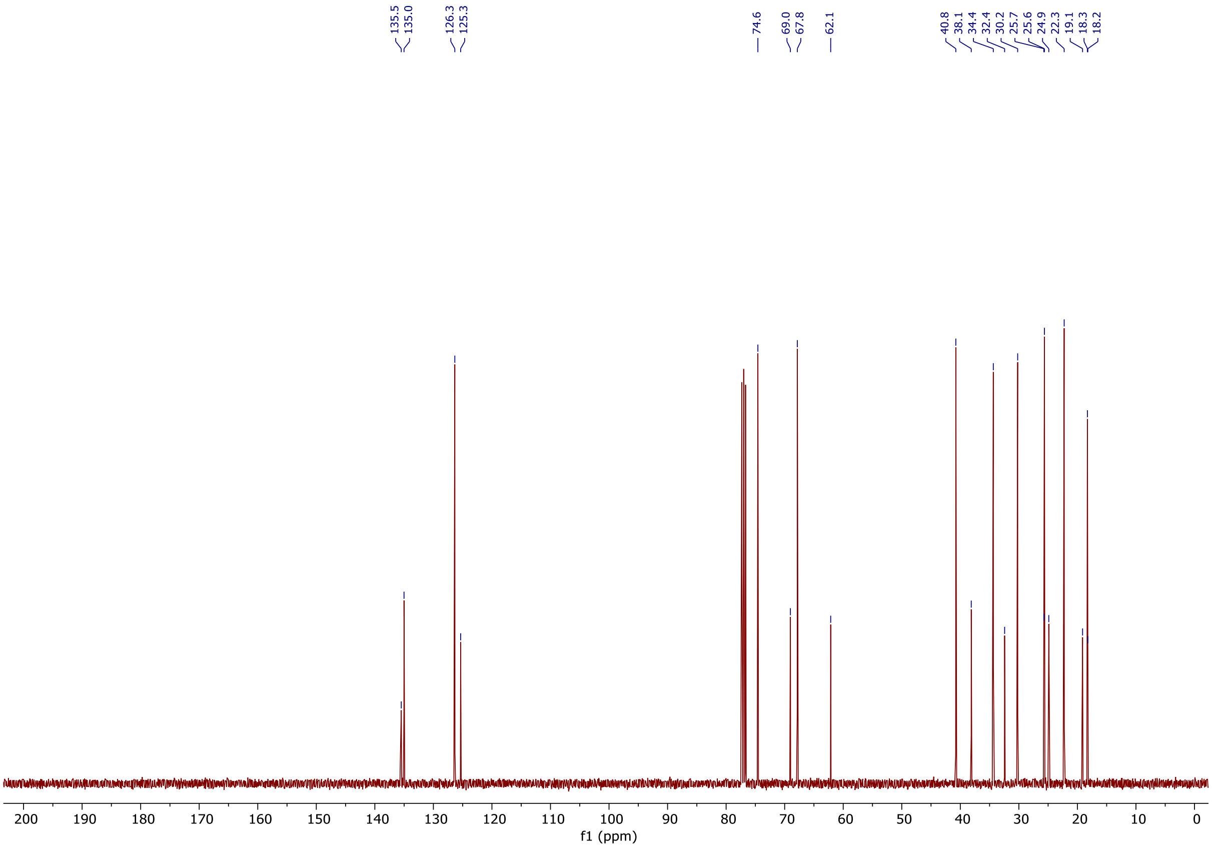

^{13}C-NMR (100 MHz, $CDCl_3$)

8 References

[1] H. Wang, S. Jiang, S. Chen, D. Li, X. Zhang, W. Shao, X. Sun, J. Xie, Z. Zhao, Q. Zhang, Y. Tian, Y. Xie, *Adv. Mater.* **2016**, *28*, 6940-6945.

[2] J. Huang, M. Klahn, X. Tian, X. Dai, J. Rabeah, V. Aladin, B. Corzilius, S. Bartling, H. Lund, N. Steinfeldt, T. Peppel, A. J. Logsdail, H. Jiao, J. Strunk, *ACS Appl. Nano Mater.* **2024**, *7*, 7442-7452.

[3] F. Lorenz, T. Peppel, M. Brasholz, J. Strunk, *ChemCatChem* **2025**, *17*, e202401847.

[4] J. J. Dong, P. Saisaha, T. G. Meinds, P. L. Alsters, E. G. Ijpeij, R. P. van Summeren, B. Mao, M. Fañanás-Mastral, J. W. de Boer, R. Hage, B. L. Feringa, W. R. Browne, *ACS Catal.* **2012**, *2*, 1087-1096.

[5] S. Doherty, J. G. Knight, J. R. Ellison, D. Weekes, R. W. Harrington, C. Hardacre, Haresh Manyar, *Green Chem.* **2012**,*14*, 925-929.

[6] K. Kamata, K. Sugahara, R. Ishimoto, S. Nojima, M. Okazaki, T. Matsumoto, N. Mizuno, *ChemCatChem* **2014**, *6*, 2327-2332.

[7] E. T. Poursaitidis, F. Trigka, C. Mantzourani, M. G. Kokotou, I. Triandafillidi, Christoforos G. Kokotos, *Eur. J. Org. Chem.* **2024**, e202400082.

[8] T. Keutz, D. Cantillo, C. O. Kappe, *Org. Lett.* **2019**, 21, 10094-10098.

[9] E. A. Mercier, C. D. Smith, M. Parvez, T. G. Back, *J. Org. Chem.* **2012**, *77*, 3508-3517.

[10] C. P. Burke, L. Shu, Y. Shi, *J. Org. Chem.* **2007**, *72*, 6320-6323.

[11] Z.-W. Zhang, H.-B. Li, J. Li, C.-C. Wang, J. Feng, Y.-H. Yang, S. Liu, *J. Org. Chem.* **2020**, *85*, 537–547.

[12] Y. Okazaki, F. Ando, J. Koketsu, *Bull. Chem. Soc. Jpn.* **2003**, *76*, 2155-2165.

[13] F. Lévesque, P. H. Seeberger, *Org. Lett.* **2011**, *13*, 5008-5011.

[14] B. M. Trost, M. R. Machacek, B. D. Faulk, *J. Am. Chem. Soc.* **2006**, *128*, 6745-6754.
